# Preparation of ^18^F-Labeled Tracers Targeting Fibroblast Activation Protein via Sulfur [^18^F]Fluoride Exchange Reaction

**DOI:** 10.3390/pharmaceutics15122749

**Published:** 2023-12-10

**Authors:** Austin Craig, Jürgen Kogler, Markus Laube, Martin Ullrich, Cornelius K. Donat, Robert Wodtke, Klaus Kopka, Sven Stadlbauer

**Affiliations:** 1 Helmholtz-Zentrum Dresden-Rossendorf, Institute of Radiopharmaceutical Cancer Research, Bautzner Landstraße 400, D-01328 Dresden, Germany; a.craig@hzdr.de (A.C.);; 2 Faculty of Chemistry and Food Chemistry, School of Science, Technische Universität Dresden, D-01062 Dresden, Germany

**Keywords:** automation, cancer-associated fibroblast, FAPI, ^18^F fluorination, positron emission tomography (PET), [^18^F]SuFEx

## Abstract

Early detection and treatment of cancers can significantly increase patient prognosis and enhance the quality of life of affected patients. The emerging significance of the tumor microenvironment (TME) as a new frontier for cancer diagnosis and therapy may be exploited by radiolabeled tracers for diagnostic imaging techniques such as positron emission tomography (PET). Cancer-associated fibroblasts (CAFs) within the TME are identified by biomarkers such as fibroblast activation protein alpha (FAPα), which are expressed on their surfaces. Targeting FAPα using small-molecule ^18^F-labeled inhibitors (FAPIs) has recently garnered significant attention for non-invasive tumor visualization using PET. Herein, two potent aryl-fluorosulfate-based FAPIs, **12** and **13,** were synthetically prepared, and their inhibition potency was determined using a fluorimetric FAP assay to be IC_50_ 9.63 and 4.17 nM, respectively. Radiofluorination was performed via the sulfur [^18^F]fluoride exchange ([^18^F]SuFEx) reaction to furnish [^18^F]**12** and [^18^F]**13** in high activity yields (AY) of 39–56% and molar activities (A_m_) between 20–55 GBq/µmol. In vitro experiments focused on the stability of the radiolabeled FAPIs after incubation with human serum, liver microsomes and liver cytosol. Preliminary PET studies of the radioligands were performed in healthy mice to investigate the in vivo biodistribution and ^18^F defluorination rate. Fast pharmacokinetics for the FAP-targeting tracers were retained and considerable bone uptake, caused by either ^18^F defluorination or radioligand accumulation, was observed. In summary, our findings demonstrate the efficiency of [^18^F]SuFEx as a radiolabeling method as well as its advantages and limitations with respect to PET tracer development.

## 1. Introduction

Positron emission tomography (PET) is a highly sensitive non-invasive imaging technique routinely utilized in clinical practice in combination with MRI or CT for the diagnosis of a plethora of human diseases [1,2,3,4,5,6]. PET imaging affords valuable and precise metabolic information on a molecular level in real time, which aids clinicians to decide on effective treatment plans for patients. Emerging biological targets for radiopharmaceuticals provide novel opportunities for enhanced tumor delineation using PET. Recently, the tumor microenvironment (TME) has gained attention as a source of PET tracer targets, owing to its inherent unique features such as the interconnection between cancer and stromal cells [7,8,9,10]. The overexpression of fibroblast activation protein alpha (FAPα) is a characteristic of cancer-associated fibroblasts (CAFs), which are located in the stroma of epithelial cells. In contrast, FAP expression in healthy tissue is relatively low [11]. 

**Scheme 1:**
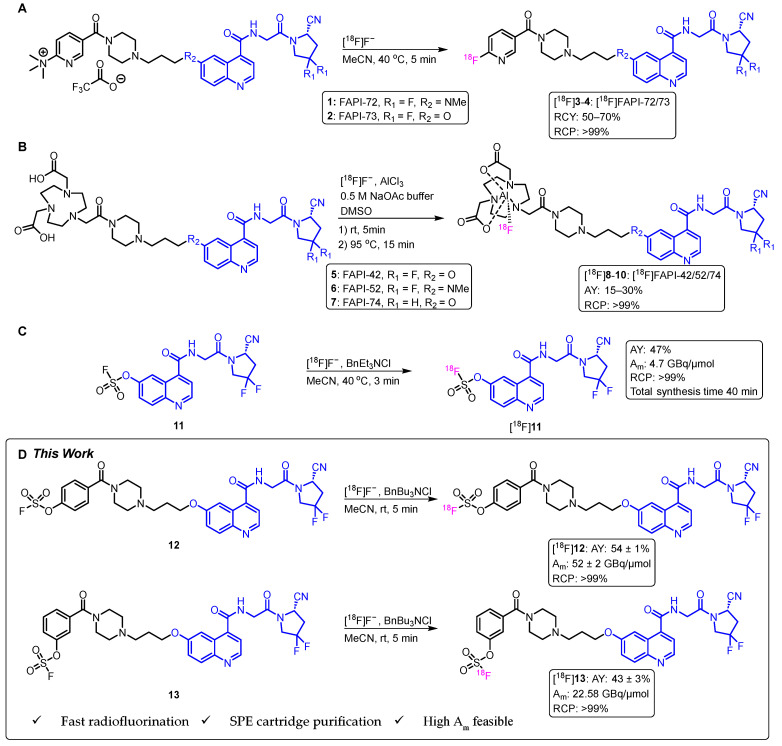
(**A**–**C**) Previous preparations of ^18^F-labeled FAPI derivatives. (**D**) This work: ^18^F-labeled FAPIs prepared via [^18^F]SuFEx reaction.

To date, several examples of radiolabeled FAP inhibitors (FAPIs) have been described [11,12,13,14]. The preparation of ^68^Ga-labeled FAPIs has the advantage of utilizing a ^68^Ge/^68^Ga generator for tracer production, thereby negating the need for an on-site cyclotron and thus reducing tracer production costs. First-in-human experiments using ^68^Ga-labeled FAPIs have provided high-contrast tumor PET images [10,11,12,14]. However, the clinical applications of ^68^Ga-labeled FAPIs are limited by the batch size of the PET tracer due to generator-defined starting activities for the production and its short half-life (68 min) [15]. Moreover, the higher positron energy associated with gallium-68 affords PET images of inferior quality compared to ^18^F-labeled radiopharmaceuticals. To overcome the limitations of ^68^Ga-based FAPIs, a series of ^18^F-labeled FAPIs have been developed. A recent work by Linder et al. provides a useful summary of ^18^F-labeled aluminum fluoride complexes and 6-fluoronicotinamide FAPI derivatives (Scheme 1A,B) [15]. The advantageous physicochemical properties of fluorine-18 are ideal for PET experiments. For example, the half-life of fluorine-18 (109.7 min), the most predominant radionuclide used in PET imaging, permits multi-step radiosynthetic protocols and tracer transport between clinical facilities [16]. Moreover, the high positron (>97% β^+^) branching and low positron energy (0.635 MeV) of fluorine-18 provide high-resolution PET images. 

The most predominant ^18^F-labeled tracer, 2-[^18^F]fluoro-2-deoxyglucose ([^18^F]FDG), has been widely implemented in clinics for the detection of various tumors using PET [17]. Despite numerous other examples of promising radiofluorinated tracers being prevalent in the literature, only ten ^18^F-labeled PET tracers have been approved by the FDA to date [18,19]. Therefore, emerging robust radiofluorination techniques that may facilitate the development of PET tracers and their translation into clinics are still highly sought after.

The development of radiofluorination methods in recent years has primarily focused on the formation of C–^18^F bonds [20,21,22,23,24,25,26,27,28,29,30,31,32,33,34]. Aside from several exceptions, radiofluorinations involving C–^18^F bond formation typically necessitate harsh reaction conditions to ensure fast ^18^F incorporation rates, which may limit their substrate scopes and practicality for clinical translation. Furthermore, fluorination strategies from larger-scale (e.g., millimolar) concentrations in organic syntheses cannot be easily adapted towards radiofluorination approaches due to the differing reactivity of fluorine-18 at low concentrations (micromolar).

The recently described sulfur [^18^F]fluoride exchange ([^18^F]SuFEx) chemistry has gained attention for the rapid preparation of ^18^F-labeled aryl-fluorosulfate (ArOSO_2_F) substrates with ultra-fast reaction times, high radiochemical yields and rapid tracer isolation via SPE purification, also demonstrated for the preparation of an ^18^F-labeled FAPI derivative (Scheme 1C) [35,36,37,38]. The ^18^F-for-^19^F isotopic exchange reaction has also been shown to produce ^18^F-labeled compounds with high molar activities (A_m_) when a low starting precursor concentration and high starting fluorine-18 activity concentration is utilized. The in vivo stability of the [^18^F]ArOSO_2_F group was also demonstrated to be sufficient for PET imaging in certain instances, yet a priori inferences regarding their stability have not been established to date. This work reports the application of the [^18^F]SuFEx chemistry towards two novel FAP-targeting radioligands and the evaluation of their suitability for non-invasive imaging using PET (Scheme 1D).

## 2. Materials and Methods

### 2.1. Materials

Unless stated otherwise, all solvents and reagents were obtained from commercial vendors and utilized without additional purification. All solvents used in experiments were of HPLC or analytical grade, with the exception of water, which was ultrapure (>18.2 MΩ cm^−1^).

### 2.2. General Information

The ^1^H, ^13^C and ^19^F NMR spectra provided were recorded on a Varian Inova-400 and *J*-values are given in Hertz (Hz). All radiochemistry experiments were performed at the Institute of Radiopharmaceutical Cancer Research, Helmholtz-Zentrum Dresden-Rossendorf (HZDR). [^18^F]Fluoride was produced via the (p,n) reaction using a TR-FLEX (ACSI) cyclotron by irradiating [^18^O]H_2_O with 18–30 MeV protons [39]. Automated radiosynthesis was carried out in a TRACERlab FX FDG (GE Healthcare) synthesis module and was performed in a hot cell. Analytical radio-(U)HPLC was performed on the following system: column Kinetex C-18 (Phenomenex Inc., Torrance, CA, USA; 50 × 2.1 mm, 1.7 µm, 100 Å), Shimadzu Nexera X2 UHPLC system (Shimadzu Corporation, Kyoto, Japan; degasser DGU-20A_3R_ and DGU-20A_5R_, pump LC-30AD, autosampler SIL-30AC, column oven CTO-20AC with two column switching valves FCV-14AH, diode array detector SPD-M30A, fluorescence detector RF-20A, γ detector Gabi Star (Raytest Isotopenmeßgeräte GmbH, Straubenhardt, Germany), communication bus module CBM-20A), eluent: (A): 0.1% trifluoroacetic acid in H_2_O, (B): MeCN. Gradient A radio-UHPLC: flow rate 0.5 mL/min, gradient (eluent A/B): *t*_0 min_ 95/5–*t*_0.3 min_ 95/5–*t*_4.5 min_ 5/95–*t*_5.5 min_ 5/95–*t*_6.0 min_ 95/5–*t*_7.5 min_ 95/5, flow rate: 0.5 mL/min. Gradient B: radio-HPLC: flow rate 1.0 mL/min, gradient (eluent A/B): *t*_0 min_ 95/5–*t*_3.0 min_ 95/5–*t*_28.0 min_ 5/95–*t*_34.0 min_ 5/95–*t*_35.0 min_ 95/5–*t*_40.0 min_ 95/5. The radiochemical conversion (RCC) based on radio-UHPLC was determined by analyzing an aliquot after dilution of the crude radiofluorination reaction mixture (MeCN) with MeCN/H_2_O (1:1) and is based on the relative peak areas of the γ detector channel of the chromatogram. The identity of the radiolabeled products was determined by associating the UV-(U)HPLC traces of the suitable unlabeled reference compounds with the radio-(U)HPLC traces of the radiolabeled products. Notably, as the concentrations of the unlabeled precursor (which is also the appropriate reference compound due to the isotopic-exchange radiolabeling mechanism of the [^18^F]SuFEx reaction) was typically sufficient for detection in the UV chromatogram, co-injection with additional reference standard was not performed. The radiochemical purity (RCP) of radiolabeled products was determined based on the integration of the peaks in the chromatogram in the radio-(U)HPLC. The isolated activity yield (AY) of a radiolabeled product is the non-decay-corrected (n.d.c.) yield given as a percentage value, which is determined by dividing the product activity at the end of synthesis (E.O.S.) by the initial starting activity and multiplying by one hundred. The molar activity (A_m_) was determined with a (U)HPLC analysis based on the injection of a known activity amount followed by determination of the injected amount of substance. For that, the UV peak area corresponding to the radiolabeled product was determined and the concentration of the non-radiolabeled “carrier” compound was calculated based on a calibration curve (See Supplementary Materials Figure S21, 5.8913 pmol o.c., and Figure S22, 22.521 pmol o.c.).

### 2.3. Preparation of Radiofluorinated FAPI Derivatives

The radiosynthetic procedure towards [^18^F]**12** and [^18^F]**13** commenced with the adsorption of [^18^F]fluoride (50–10,000 MBq) on an anion-exchange resin (QMA light carbonate cartridge, Waters Corp., Milford, MA, USA) followed by washing with dry MeOH (0.7 mL) from the male side and elution of ^18^F with a solution of either 3.0 mg (9.6 µmol) BnBu_3_NCl, 3.0 mg (13.1 µmol) BnEt_3_NCl or 1.0 mg (5.2 µmol) Et_4_NHCO_3_ in 0.7 mL MeOH from the female side (Scheme 1D). The methanolic solution was removed under vacuum at 70 °C for 5 min. The radiolabeling precursors (**12** or **13,** 0.1 mg, 0.145 µmol) in MeCN (0.5 mL) were added to the cooled reaction vessel and allowed to react for 5 min at room temperature without stirring. The reaction mixture containing the crude radiofluorinated product ([^18^F]**12** or [^18^F]**13**) was diluted with (5 mL) H_2_O and passed through an HLB SPE cartridge. The HLB cartridge (Waters Corp.) was washed with water (10 mL) and eluted with 2 mL EtOH to furnish either [^18^F]**12** at an activity yield (AY) of 55 ± 1% (*n* = 3) or [^18^F]**13** at an activity yield of 43 ± 3% (*n* = 3) with a radiochemical purity of >95%. The product solution was further diluted with 0.9% NaCl aqueous solution to obtain a final product containing 10% of EtOH (*v*/*v*). The final products were analyzed using analytical radio-HPLC for product identification. The RCC determination was carried out following the quenching of the reaction mixture with (100 µL) H_2_O, taking an aliquot (5–15 µL) of the crude reaction mixture and adding the mixture to a sample of (200 µL) H_2_O/MeCN (1:1, *v*/*v*), and analyzed using radio-UHPLC. The RCC value was determined by integrating the radiolabeled product peak area, dividing this value by the total integrated peak area values and multiplying the value by one hundred. The AY was obtained by dividing the measured activity of the product by the measured starting activity of [^18^F]F^−^ prior to radiosynthesis and multiplying by one hundred [40]. Unless noted otherwise, each experiment was performed in triplicate. Calibration curves (see Supplementary Materials, Figures S21 and S22) were constructed from the peak areas in the UV channel of the UHPLC chromatograms of a series of non-radiolabeled products (e.g., **12**) with known concentrations, in order to determine exemplarily the carrier concentration in the final radiolabeled product mixture.

### 2.4. Radiosynthesis Automation

The automated radiosynthesis of [^18^F]**12** was carried out in a TRACERlab FX FDG synthesis module (GE Healthcare, Waukesha, WI, USA). Approximately 6–60 GBq of aqueous non-carrier-added (n.c.a.) [^18^F]fluoride was trapped on a QMA light carbonate cartridge (Waters Corp., Position 1), washed with 2.5 mL MeOH (V1) and eluted from the male side with Et_4_NHCO_3_ (1.0 mg, 5.2 µmol, V2) in 700 µL MeOH.

Evaporation of the MeOH was carried out at 70 °C for 5 min under a vacuum. After cooling the reaction vessel to 23 °C, **12** (0.1 mg, 0.145 µmol) in 0.5 mL MeCN (entry V3) was transferred to the reactor. The reaction was performed for 5 min at 23 °C. Thereafter, the reaction was quenched with the addition of 7 mL H_2_O into the reactor (V6), and the resulting solvent mixture was loaded onto a preconditioned HLB cartridge (Waters Corp., position 1—HLB). Thereafter, the HLB cartridge was washed with 12 mL H_2_O (V7). Finally, the elution of the trapped product [^18^F]**12** from the HLB cartridge was carried out using 2 mL EtOH (V8) into the product vial. The purified radiotracer was diluted in isotonic saline (0.9% NaCl) to obtain a final product containing 10% of EtOH (*v*/*v*). The final product was analyzed using radio-HPLC for product identification. Overall, the implementation of the radiosynthetic protocol into an automated radiosynthesizer furnished the desired product [^18^F]**12** within 60 min with an AY of 11 ± 1% (*n* = 3).

### 2.5. Determination of Lipophilicity

The LogD_7.4_ determination of [^18^F]**12** was determined in a similar manner as previously described and proceeded as follows [41]. [^18^F]**12** (1 μL, ca. 0.5 MBq) was pipetted into an Eppendorf tube containing 600 μL of *n*-octanol (presaturated with phosphate-buffered saline) and 600 μL of phosphate-buffered saline buffer (0.02 M, pH = 7.4). The tube was vortexed for 15 min at room temperature, and the two phases were separated by centrifugation at 5000 rpm for 3 min. An aliquot (400 µL) from the *n*-octanol layer was pipetted in a vial containing phosphate-buffered saline (400 µL), the sample mixture was vortexed for 15 min at room temperature and the two phases were separated by centrifugation at 5000 rpm for 3 min. Aliquots (250 µL) from each phase were taken and measured with an automatic γ-counter after subtracting the background activity. The transfer of the aqueous phase requires particular care, as described by Linclau et al. [42]. The partition coefficient was calculated for the acquired samples using Equation (1).

(1) $$logD_{7.4}=log\;\frac{counts\;per\;minute\;(octanol)}{counts\;per\;minute\;(phosphate\;buffer)}$$

Equation (1) for lipophilicity (LogD) determination.

### 2.6. Serum Stability Assay

Either [^18^F]**12** or [^18^F]**13** (20–40 MBq) in 40 µL EtOH aliquots was incubated with 360 µL human serum (Sigma Aldrich, Darmstadt, Germany) at 37 °C. After specific time intervals (5–120 min), 40 µL samples were taken from the serum incubation mixture, added to 80 µL “Supersol” solubilizing agent (see Supplementary Materials Table S6) and cooled over ice for 2 min. Thereafter, the cooled mixture was centrifuged at 4 °C (15,000 rpm) for 3 min and 80 µL of the supernatant solution was monitored with radio-UHPLC. A gradient of 5% to 45% acetonitrile in 7.5 min was used for enhanced separation.

### 2.7. Liver Microsome Assay

Microsome experiments with [^18^F]**12** in the presence of NADPH were performed according to the procedure recently described by us with slight modifications [43,44]. Incubations had a final volume of 250 µL. The radiotracer dissolved in EtOH (4 µL; 5 MBq/µL) was diluted with PBS (496 µL, 0.8% EtOH). This radiotracer solution (100 µL, 0.32% EtOH and 16 MBq/mL or 1.6 µM final) was mixed with PBS (112.5 µL) and human liver microsomes (12.5 µL of 20 mg/mL stock; 1 mg/mL final; Gibco™ Cat. No. HMMCPL, Lot. No. PL050E-B) in a 1.5 mL Eppendorf tube and the mixture was warmed for 5 min at 37 °C. Subsequently, NADPH (25 µL of freshly prepared 20 mM solution in PBS, 2 mM final) was added and the mixture was incubated at 37 °C. As a control incubation, NADPH was omitted and replaced by PBS. After distinct time points (10, 20, 30 and 60 min), an aliquot (40 µL) was withdrawn and added to ice-cooled CH_3_CN (160 µL). The mixture was vortexed for 30 s, stored on ice for 4 min and centrifuged (5 min at 15,000 rpm). The resulting supernatant was used for radio-HPLC analysis using the Shimadzu system described in Section 2.2 with the following conditions. A C_18_ column Kinetex^®^ from Phenomenex (5 µm, 100 Å, LC Column 250 × 4.6 mm) served as stationary phase. The eluent consisted of (A) 0.1% trifluoroacetic acid in H_2_O and (B) MeCN; flow rate 1 mL/min; elution profile A/B: *t*_0 min_ 70/30–*t*_10.0 min_ 70/30–*t*_11.0 min_ 5/95–*t*_16.0 min_ 5/95–*t*_17.0 min_ 70/30–*t*_22.0 min_ 70/30.

Testosterone (40 µM final) was used as a positive control for the activity of the HLM and was separately incubated under the conditions described above (0% EtOH final). The metabolization was analyzed at distinct time points (10, 20, 30, 40 and 60 min) with the HPLC system specified above (254 nm, elution profile A/B: *t*_0 min_ 55/45–*t*_10.0 min_ 55/45–*t*_11.0 min_ 5/95–*t*_16.0 min_ 5/95–*t*_17.0 min_ 55/45–*t*_25.0 min_ 55/45) and was completed after >30 min.

### 2.8. Liver Cytosol Assay

Liver cytosol experiments with [^18^F]**12** were performed according to reported procedures [45,46]. Incubations had a final volume of 250 µL. PBS (125 µL) and human (HLC, Gibco™ Cat. No. HMCYPL; Lot. No. PL028-J) or rat (RLC, Gibco™ Cat. No. RTCYPL; Lot. No. RT062-B) liver cytosol (25 µL of 20 mg/mL stock; 2 mg/mL final) were mixed in a 1.5 mL Eppendorf tube and the mixture was warmed for 5 min at 37 °C. Subsequently, the radiotracer solution (100 µL, 1% EtOH and 21 MBq/mL or 2.1 µM final) was added and the mixture was incubated at 37 °C. After distinct time points (10, 30 and 60 min), an aliquot (40 µL) was withdrawn and added to ice-cooled CH_3_CN (160 µL). The mixture was vortexed for 30 s, stored on ice for 4 min and centrifuged (5 min at 15,000 rpm). The resulting supernatant was used for radio-HPLC analysis using the conditions described in Section 2.3.

Vanilin (40 µM final) was used as positive control for the activity of the liver cytosols and was separately incubated under the conditions described above (1% EtOH final). Its metabolization and the simultaneous formation of vanilic acid was analyzed at distinct time points (10, 30 and 60 min) with the HPLC system specified above (280 nm, elution profile A/B: *t*_0 min_ 78/22–*t*_10.0 min_ 78/22–*t*_11.0 min_ 5/95–*t*_16.0 min_ 5/95–*t*_17.0 min_ 78/22–*t*_22.0 min_ 78/22). More than 80% of the vanilin had been degraded after 60 min in the presence of HLC, while around 60% of the vanilin had been degraded with RLC. There was no change in the degradation rate with and without 1% EtOH in the incubation mixture.

### 2.9. FAP Fluorogenic Assay

To determine the inhibitory capacity, a commercial fluorogenic FAP assay was used (BPS Bioscience, San Diego, CA, USA, #80210) according to the manufacturer’s instructions. All tested compounds (**12**, **13** and FAPI-04) were diluted in DMSO. FAPI-04 was synthetically prepared in a similar manner to that previously reported [12] along with comparable purity and characterization. On the day of testing, a 1:10 dilution in the supplied DPP assay buffer was performed for all compounds. The final mix on the 96-well plate yielded 10 concentrations of each compound, ranging from 1 µM to 50 pM with 1% DMSO (*v*/*v*), carried out in duplicates. The fluorogenic peptide substrate (Ala-Pro-AMC dipeptide) was added and the reaction started with the addition of the human recombinant FAP (25 ng/µL). Immediately, the plate was loaded into a multilabel plate reader (Biotek Cytation 5, Agilent, Bellevue, WA, USA) and each well was measured at an excitation wavelength window of 360 ± 20 nm and an emission wavelength window of 450 ± 20 nm. The fluorescence intensity was measured over 15 min in intervals of 15 s, resulting in 61 data points per curve. Controls included blanks (omission of substrate, enzyme and inhibitor), negative (omission of enzyme) and positive controls (omission of inhibitor). The recorded time courses of the type (RFU − RFU0) = f(t) were analyzed with linear regression of the experimental data over the entire measurement period. The IC_50_ value, which is equal to the inhibitor amount that causes 50% inhibition, and the Hill slope, nH, were calculated according to Equation (2), with “bottom” and “top” representing the lower and upper plateaus of the sigmoid dose−response curve, respectively [44].

(2) $$rate=Bottom+\frac{(Top-Bottom)\times {\left[I\right]}^{nH}}{{\left[I\right]}^{nH}+{{IC}_{50}}^{nH}}$$

Equation (2) for analyzing the sigmoid dose–response curve.

### 2.10. PET Imaging Experiments

All animal experiments were carried out according to the guidelines of the German Regulation for Animal Welfare. The protocol was approved by the local Ethical Committee for Animal Experiments. Small-animal PET was performed using the nanoScan^®^ PET/CT (Mediso Medical Imaging Systems, Budapest, Hungary). Each animal (BALB/cJRj mice, *n* = 2) received an intravenous injection of 10 MBq [^18^F]**12** delivered in 0.15 mL of 0.154 mol/L NaCl_aq_ through a tail vein catheter. Emission of annihilation photons was recorded continuously for 2 h. A corresponding X-ray CT image was recorded and used for anatomical referencing and attenuation correction. List-mode data were sorted into sinograms with 36 frames (15 × 10 s, 5 × 30 s, 5 × 60 s, 4 × 300 s, 3 × 600 s, 4 × 900 s) and reconstructed using the Tera-Tomo™ 3D algorithm, applying corrections for decay, scatter and attenuation. Images were post-processed and analyzed using ROVER (ABX, Radeberg, Germany) and displayed as maximum-intensity projections (MIP) at indicated the time points and scaling. Three-dimensional regions of interest (ROI) were created, applying fixed contrast thresholds for delineation of blood (heart content, 80%), liver (80%), kidneys (50%), bone (knee and shoulder joints, 50%), gall bladder and intestine (5%) and urinary bladder (10%). Organ-specific activity concentrations were determined as ROI-averaged standardized uptake values at mid-frame time (SUV_mean_). Time–activity curves were drawn using Prism 9.0 (GraphPad Software, La Jolla, CA, USA) and the activity retention within each organ was reported as the area under the curve (AUC). Excreted activity fractions were determined as the percent of the initially administered activity dose (%ID).

## 3. Results

### 3.1. Organic Synthesis of Radiolabeling Precursors

**Scheme 2:**
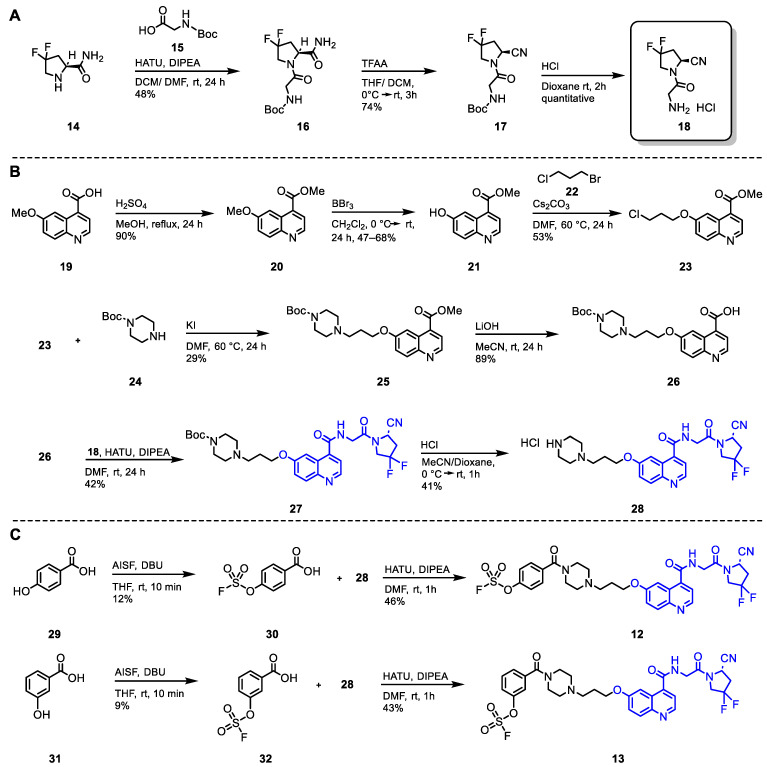
(**A**) Preparation of difluorinated proline building block **18**; (**B**) synthetic route towards FAPI intermediate **28** and (**C**) preparation of radiolabeling precursors **12** and **13**.

The intermediate building blocks **18** and **28** were accessed via a previously described synthetic route [12,47] and obtained in overall yields of 36% and 1%, respectively (Scheme 2A,B). The fluorosulfonation of the commercially available phenols **29** and **31** was achieved using AISF (4-(acetylamino)phenyl]imidodisulfuryl difluoride) and DBU as a base in THF to afford aryl-fluorosulfates **30** and **32** in 9% and 12% yields [48], respectively (Scheme 2C) [49]. Thereafter, aryl-fluorosulfates **30** and **32** were coupled with **28** using HATU and DIPEA in DMF to furnish the final compounds **12** and **13** in 46% and 43% yields, respectively. Both **12** and **13** serve as radiolabeling precursors and reference compounds for product identification in the radiosynthesis via [^18^F]SuFEx.

### 3.2. Fluorogenic FAP Assays

**Figure 1:**
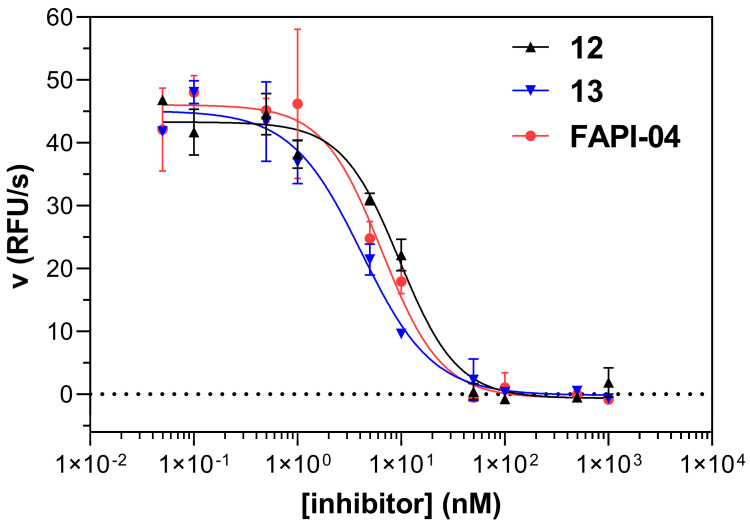
Inhibition of human recombinant FAPα enzyme activity by compounds **12**, **13** and FAPI-04. Data shown are mean values (±SD) of one experiment for each compound, which was performed in duplicate.

Employing a commercially available enzyme assay, compounds **12** and **13** along with a reference (FAPI-04) were probed for their potency to inhibit recombinant human FAP enzyme activity (Figure 1). Spanning a range of compound concentrations (50 pM–1 µM), fluorescence intensity changes were followed over 15 min. For all compounds, a linear response in relative fluorescence units (RFU) was observed, with the slope being determined by the concentration of the inhibitor. This is exemplified by the response of FAPI-04, provided in the supporting information.

By using this approach, compounds **12** and **13** were found to inhibit human FAP with IC_50_ values of 9.63 and 4.17 nM, respectively (Table 1). These data are comparable to the IC_50_ value for the reference compound FAPI-04 determined in the same assay (6.55 nM).

### 3.3. Preparation of ^18^F-Labeled Tracers Targeting Fibroblast Activation Protein

The optimization of the radiofluorination was performed utilizing a selection of phase transfer agents (PTA) in manual radiosyntheses. This covered ^18^F recovery as a measure of the separation of [^18^F]fluoride from target water using the minimalist approach, as well as the [^18^F]SuFEx reaction at room temperature by measuring the RCC value at three time points (Table 2) [50]. Et_4_NHCO_3_ was chosen as a very common base in [^18^F]fluorinations and as starting point for the developments, while BnBu_3_NCl and BnEt_3_NCl were chosen as more novel PTAs, as introduced by Neumaier et al. for [^18^F]SuFEx chemistry [36]. For all three PTAs, high ^18^F recovery (>90%) from the QMA was found (Table 2). Radiofluorination was found to proceed fast and within 5 min with all three PTAs. However, considerable differences were found for isolated activity yields (AY) comparing Et_4_NHCO_3_, a more basic PTA, with the chloride salts BnBu_3_NCl and BnEt_3_NCl (neutral PTAs). The basicity of Et_4_NHCO_3_ was found to lead to the decomposition of both [^18^F]**12** and [^18^F]**13**, as reflected by the significant RCC range between the radiolabeling experiments and lower AY. In a direct comparison, BnBu_3_NCl was determined to be the optimum PTA for both ^18^F recovery (97 ± 1%) and the final AY. Furthermore, the *para*-derivative [^18^F]**12** was found to provide a higher RCC compared to its *meta*-analog [^18^F]**13** with all three PTAs. Under optimized conditions for manual radiosynthesis, [^18^F]**12** and [^18^F]**13** could be obtained in 55 ± 1% and 43 ± 3% activity yields, respectively, using BnBu_3_NCl. Under these conditions and a starting activity of ca. 10 GBq, molar activity (A_m_) values of 19.7 GBq/µmol for [^18^F]**12** and 22.6 GBq/µmol for [^18^F]**13** were observed. The LogD_7.4_ value for [^18^F]**12** was determined by partitioning between phosphate-buffered saline at pH 7.4 (PBS) and octanol to be 1.81.

**Figure 2:**
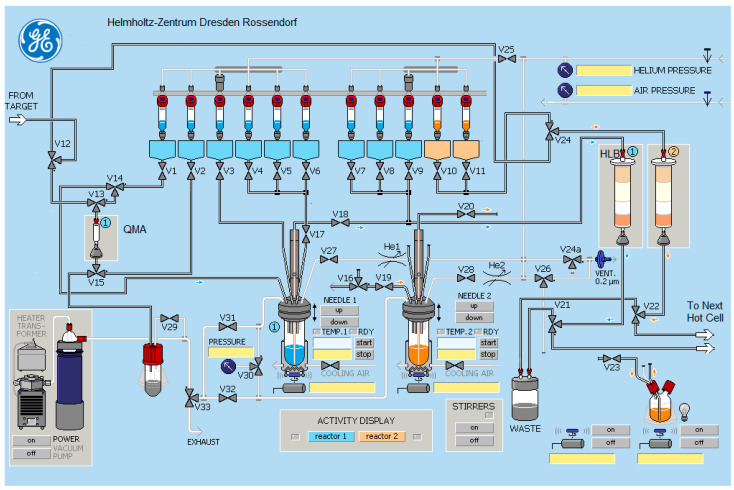
Overview of the synthesis module TRACERlab FX FDG for the radiosynthesis of [^18^F]**12**. (1, QMA) QMA light carbonate cartridge, (V1) 2.5 mL MeOH, (V2) BnEt_3_NCl (3 mg in 1.5 mL MeOH), (**V3**) radiolabeling precursor (0.1 mg, 0.145 µmol of **12** in 500 µL MeCN), (V6) 7.0 mL H_2_O, (V7) 7.0 mL H_2_O, (V8) 2.0 mL EtOH, (1, HLB) Sep-Pak^®^ HLB, product vial was placed in neighboring hot cell.

As part of the work process, the radiosynthesis using Et_4_NHCO_3_ as base was transferred to an automated radiosynthesizer (Figure 2) including separation of [^18^F]fluoride from target water, solvent evaporation, [^18^F]SuFEx and final SPE purification using an HLB cartridge. Accordingly, [^18^F]**12** was obtained in 11 ± 1% (*n* = 3) AY starting from 47.4 GBq furnishing the radiotracer in A_m_ of 53 ± 2 GBq/µmol.

### 3.4. Stability Studies of Radiolabeled Compounds

**Figure 3:**
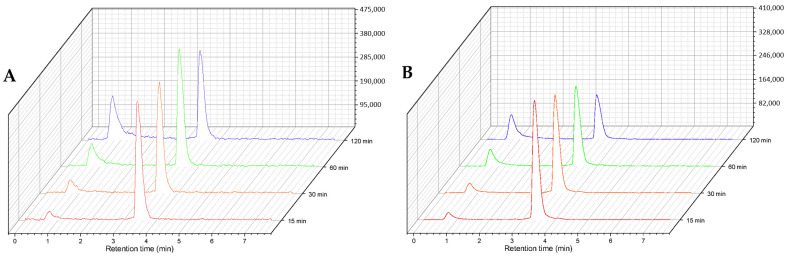
(**A**) Stability studies of [^18^F]**12** in human serum at 37 °C. (**B**) Stability studies of [^18^F]**13** in human serum at 37 °C.

The radiochemical purity and identity of [^18^F]**12** and [^18^F]**13** was analyzed with radio-HPLC. The comparison to the non-radioactive references **12** and **13** confirmed their identities and revealed that a radiochemical purity (RCP) of >95% was obtained. Prior to a biological evaluation, preliminary stability studies of [^18^F]**12** and [^18^F]**13** were performed in human serum at 37 °C (Figure 3A,B). The stability studies were performed by taking an aliquot of the radiolabeled product solution and incubating the product mixture in the desired solvent or human serum at 37 °C for the given time points. In both cases, decomposition of [^18^F]**12** and [^18^F]**13** was evident from the first peak in the radioactive channel (t_R_: 0.6–1.0), most likely due to the release of [^18^F]fluoride, which could be detected using UHPLC analysis from the first time point of 15 min and continued until 120 min. After 60 min, approximately 20% of [^18^F]**12** and 19% of [^18^F]**13** had been degraded; following a 120 min incubation, approximately 38% of both [^18^F]**12** and [^18^F]**13** had been degraded. For comparison, both [^18^F]**12** and [^18^F]**13** have been shown to be stable after 120 min in PBS (pH 7.4) (see Supplementary Materials Figures S19 and S20).

### 3.5. Liver Microsome Experiments

**Figure 4:**
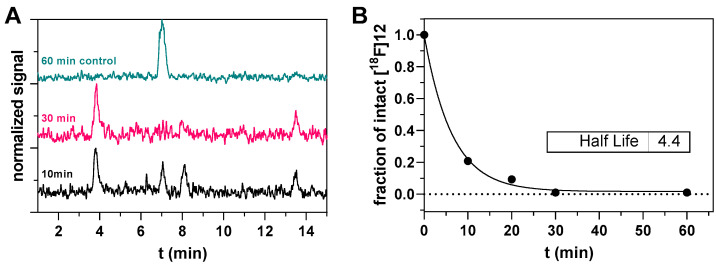
(**A**) Radio-HPLC chromatograms of [^18^F**]12** after incubation with human liver microsomes (HLM) for the indicated time periods. For “60 min control”, NADPH was omitted in the incubations. (**B**) Plot of fraction of intact [^18^F]**12** determined with HPLC analysis vs. time including nonlinear regression according to one-phase decay. The calculated half-time of [^18^F]**12** toward HLM is given in min in the box. Conditions: 10 mM PBS (pH 7.4), 1 mg/mL HLM, 2 mM NADPH, 16 MBq/mL or 1.6 µM [^18^F]**12**, 0.32% EtOH (*v*/*v*).

[^18^F]**12** was furthermore subjected to incubation in the presence of human liver microsomes (HLM) under oxidative conditions (NADPH) and human (HLC) and rat liver cytosol (RLC) (Figure 4A). Liver microsome stability studies have shown that [^18^F]**12** appeared to be largely stable toward HLC and RLC up to 60 min (see Supplementary Materials Figure S23). However, a time-dependent degradation was observed toward HLM, revealing a half-time of only 4.4 min (Figure 4B). At least three radiolabeled metabolites could be detected using radio-RP-HPLC analysis, with two of them eluting even at greater retention times than the parent radiotracer. The control experiment was performed with the omission of NADPH, and it was found that [^18^F]**12** was apparently stable in the presence of HLM without oxidative conditions. Of note, liver microsome experiments were analyzed with radio-HPLC, where [^18^F]fluoride and, hence, the release of [^18^F]fluoride cannot be reliably detected.

### 3.6. Distribution of [^18^F]12 in Mice

**Figure 5:**
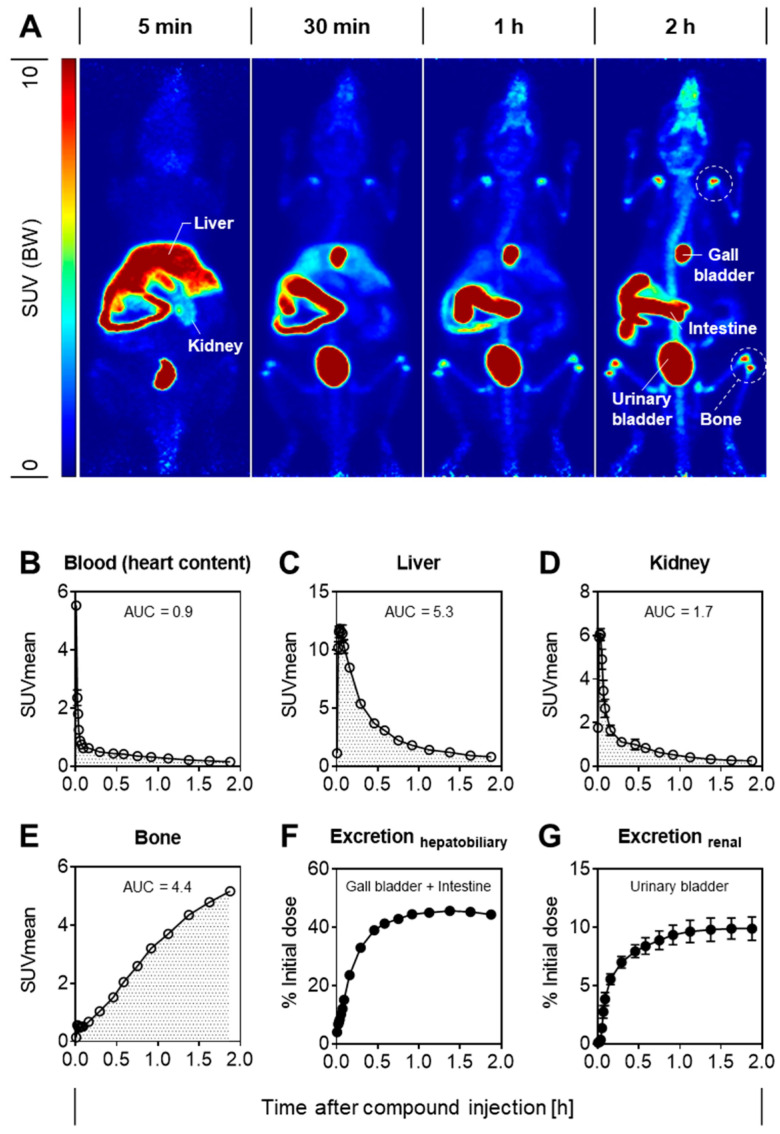
Imaging of [^18^F]12 distribution in BALB/cJRj mice; (**A**) PET images presented as maximum intensity projections (prone view) at different time points after compound injection; (**B**–**E**) kinetics of organ-specific activity concentrations presented as region-averaged standardized uptake values (SUV_mean_); areas under curve (AUC) indicate the overall activity retention within 2 h of observation; (**F**,**G**) kinetics of hepatobiliary and renal excretion presented as percent of initially administered activity dose.

As exemplarily investigated for compound [^18^F]**12**, small-animal PET showed the in vivo distribution of the radiofluorinated compound and its putative ^18^F-containing metabolites in mice within 2 h after injection (Figure 5A). Approximately 90% of the initial activity concentration in blood (SUV_mean_ of heart content) was cleared within 5 min after compound injection (Figure 5B). Compound [^18^F]**12** showed fast liver and kidney uptake with maximum activity concentrations 2 min after injection followed by 90% clearance from the liver within 82 min and from kidneys within 45 min **(**Figure 5C,D). Furthermore, in vivo administration of [^18^F]**12** in mice was followed by continuously increasing activity concentrations in bones, most likely due to the release of [^18^F]fluoride during metabolic turnover (Figure 5E).

Following [^18^F]**12** injection, distribution and metabolic turnover in mice, the major fraction of the initially delivered activity dose was excreted via the hepatobiliary pathway (45%), as determined from its retention in gall bladder and intestine (Figure 5F). A considerably smaller fraction was excreted via the renal pathway (10%), as determined from its retention in urinary bladder (Figure 5G).

## 4. Discussion

A novel class of ^18^F-labeled tracers targeting FAP has been accessed via ultra-fast [^18^F]SuFEx chemistry. Despite the introduction of an aryl-fluorosulfate in compounds **12** and **13,** both compounds retained their excellent inhibitory capacity compared to previous FAP inhibitors. The facile synthetic method towards radiolabeling precursors **12** and **13** and the highly efficient [^18^F]SuFEx radiofluorination approach allowed rapid access to the radiolabeled compounds [^18^F]**12** and [^18^F]**13** under mild radiolabeling conditions. In the context of existing ^18^F-fluorination strategies, distinct advantages of the [^18^F]SuFEx radiolabeling protocol include the omission of metal additives (e.g., Cu-mediator complexes), the furnishing of structurally diverse radiofluorinated compounds in high AYs and the simple radiosynthesis translation into commercially available radiosynthesizers. Additionally, the synthetic preparation of a cold reference compound for radiolabeled product confirmation via analytical radio-HPLC co-injection is not required, as the radiolabeling precursor may fulfil this purpose. Upon harnessing the [^18^F]SuFEx chemistry, to the best of our knowledge, our work presents one of the highest-yielding radiosynthesis routes to an ^18^F-labeled FAPI ([^18^F]**12**, 55 ± 1% AY) to date [15,36]. Manual radiosynthesis of both [^18^F]**12** and [^18^F]**13** required 25 min preparation time, with both radioligands accessed in >95% RCP. The preparation of [^18^F]**12** using the TRACERlab FX FDG synthesis module required a preparation time of 50 min and it was obtained at an AY of 11 ± 1% (*n* = 3) and with >95% RCP.

The molar activity (A_m_) of radiotracers is an important value for PET imaging, as target-site occupancy and unwanted toxic effects of injected “cold” products can be detrimental for furnishing high-resolution PET images and for ensuring patient safety. Thus, radiofluorinated products with high A_m_ values are advantageous, as they minimize the concentration of unlabeled “carrier” compounds being introduced into a patient. As mentioned previously, the A_m_ value for ^18^F-for-^19^F isotopic-exchange reactions such as the [^18^F]SuFEx reaction is of particular importance, as the impossibility of separating the radiolabeling precursor (e.g., **12**) from the radiolabeled product (e.g., [^18^F]**12**) can prove a challenge for radiochemists [51]. Fortunately, high A_m_ values for [^18^F]**12** were afforded in both manual (A_m_: 19.7 GBq/µmol from a starting activity of 10.4 GBq) and automated (A_m_: 53 ± 2 GBq/µmol from a starting activity of with 47.4 GBq) radiosyntheses. The relatively high A_m_ values were achieved by employing a relatively low concentration (0.1 mg, 0.145 µmol) of the radiolabeling precursors and commencing the radiosynthesis with a high concentration of [^18^F]fluoride, as previously described [36].

In this work, we sought to examine whether the influence of the position of the fluorosulfate group on the aromatic ring of the radioligand plays a role in (i) the AY of the final product and (ii) the overall stability of the radioligand. As of yet, a practical set of guidelines providing information on the stability of the radiofluorinated aryl-fluorosulfate ([^18^F]ArOSO_2_F) motif has not been fully established. Studies have suggested that the stability of the aryl-fluorosulfate motif may be dependent on the biovector bearing the aryl-fluorosulfate [35,36] and on the presence of activating groups on the aromatic ring bearing the fluorosulfate [36]. Neumaier et al. stated that “highly electron deficient radiolabeled aryl-fluorosulfates are not sufficiently stable for in vivo applications” due to rapid tracer defluorination (arising from nucleophilic displacement of the [^18^F]fluorosulfate) [36]. Moreover, the ability of the fluorosulfate (-OSO_2_F) group to potentially act as a chemical warhead in a similar manner to a fluorosulfonate (-SO_2_F) motif may present additional challenges [52,53], depending on the metabolic pathway of the radioligand. The exposure of an ^18^F-labeled compound bearing a fluorosulfate to proteins with a particular orientation will result in defluorination via nucleophilic displacement of the [^18^F]fluorosulfate, reflected by the accumulation of fluorine-18 in the bones and joints in a resulting PET image. Although the formation of a covalent bond of a radioligand to a target protein may provide insight into the target binding, this phenomenon is largely detrimental for PET imaging purposes using radiofluorinated aryl-fluorosulfates [54]. Therefore, information relating to the defluorination rates of PET tracers prepared using [^18^F]SuFEx radiolabeling approaches is of high significance for future radioligand design.

Extensive stability studies of the aryl-fluorosulfate group have been carried out in the seminal work by the group of Wu et al. [35], where radiolabeled aryl-fluorosulfates were incubated with nucleophilic amino acids at various pH ranges and in the presence of oxidizing reagents. However, as the later work of Neumaier et al. discussed, the analytical method initially reported by the group of Wu using a silica-based SunFire^®^ C_18_ HPLC column with an acidic mobile phase (10–100% MeCN in 0.5% TFA) for the determination of radiochemical conversion (RCC) and stability studies was not ideal, as [^18^F]HF formed following defluorination and protonation from the acidic media may be adsorbed on the HPLC column [36]. Ultimately, this would give the false impression of higher RCC values of the radiolabeled products and would additionally overestimate the stability of the ^18^F-labeled products. Attempts to circumvent the instability of aryl-fluorosulfates may be employed upon the utilization of aryl-sulfamoyl fluorides (Ar-N-SO_2_-F) as alternative radiolabeling groups [38,55,56], although extensive stability studies of radiolabeled aryl-sulfamoyl fluorides have not yet been reported.

The results displayed in Table 2 show higher RCC and AY values for compound [^18^F]**12** compared to [^18^F]**13**. Enhanced ^18^F-fluorination of [^18^F]**12** was evident within 30 s and consistent over 5 min with all PTAs evaluated. The higher ^18^F fluorination rates for [^18^F]**12** may be due to the electron-withdrawing effect of the amide group in the *para*-position. Various PTAs were screened to evaluate their effect on RCC and on the stability of the resulting radioligands. In contrast to the typically employed K_2_CO_3_/K_2.2.2._ elution methods [57,58,59], Et_4_NHCO_3_, BnEt_3_NCl and BnBu_3_NCl were chosen as advantageous alternatives in order to circumvent time-consuming azeotropic drying and to minimize product losses to decomposition under basic conditions [36]. It was found that BnBu_3_NCl afforded the highest ^18^F recovery following elution (97 ± 1%, *n* = 6) and furnished both [^18^F]**12** and [^18^F]**13** in the highest AYs of 55 ± 1% and 43 ± 3%, respectively (Table 1).

In addition, we performed a series of stability studies of compounds [^18^F]**12** and [^18^F]**13** in EtOH, phosphate-buffered saline (PBS, pH 7.4) and human serum (37 °C) to provide an indication of the rates of in vivo defluorination. The stability studies indicated that both [^18^F]**12** and [^18^F]**13** are stable in EtOH and PBS for 120 min, but show clear signs of instability after only 15 min in human serum at 37 °C (Figure 3A,B). These findings correlate with the unwanted accumulation of activity in the bones and joints obtained during PET imaging experiments in a healthy mice (Figure 5A,B). Furthermore, the SUV_bone_ uptake was shown to be relatively higher for [^18^F]**12** compared to [^18^F]**13** (Figure 5), which would support the theory that radiolabeled aryl-fluorosulfate PET tracers with electron-deficient aromatic systems are disadvantageous for imaging applications [36].

However, the relatively higher lipophilicity of the ^18^F-labeled compounds ([^18^F]**12** Log D_7.4_: 1.81) may also be responsible for the high rate of ^18^F defluorination, as the higher lipophilicity of [^18^F]**12** and [^18^F]**13** is shown to promote excretion via the liver compared to the kidney-excretion pattern observed for more hydrophilic FAPI derivatives (Scheme 1B, [^18^F]**8**–**10**) [15]. Liver microsome stability studies have demonstrated that [^18^F]**12** is mostly stable toward both HLC and RLC for up to 1 h, but is rapidly degraded upon treatment with HML (t_1/2_ of 4.4 min). Jansen et al. described the sufficient stability of FAPIs with a (4-quinolinoyl)-glycyl-2-cyanopyrrolidine scaffold in microsome preparations, with half-lives greater than 6 h (even 24 h) [47,60]. The signals of the radiometabolites in the radioactivity channels provide evidence that the radiolabeled fluorosulfate motif is still intact, and would therefore indicate that the radiometabolites observed herein originate from transformations at the benzoyl-piperazine-alkyl moiety. These transformations might include hydroxylation at the piperazine ring and probably also *N*-oxygenation at the tertiary amine by FMOs, which are also present in microsome preparations [61]. As the quinoline system is a known substrate motif for aldehyde oxidase (AO), which can lead to oxidation in *ortho*- and/or *para*-position to the nitrogen, we studied stability in liver cytosols, which are a source of AO [62]. Interestingly, [^18^F]**12** was found not to be a substrate of AO.

Taken together, our findings suggest the unsuitability of the radiolabeled aryl-fluorosulfates [^18^F]**12** and [^18^F]**13** for FAPI-PET imaging and indicate that alternating the substitution pattern of the aryl-fluorosulfate has slight advantages regarding stability. Notably, Toms et al. have shown that a high amount of activity concentration in the bones and joints of mice was also evident following the radiofluorination of a FAPI derivative via [^18^F]fluoroglycosylation [63], although this was shown to be reduced following appropriate blocking ([^18^F]FGlc-FAPI: 6.0 ± 2.5% ID/g vs. blocking: 0.36 ± 0.06% ID/g) with a non-radiolabeled FAP-targeting alkyne derivative. Due to the relatively strong [^18^F]C-F bond present in the [^18^F]fluoroglycosylation radiolabeling approach, it is unlikely that defluorination of the radiolabeled compound occurred, and this may indicate that the intact radioligand is accumulating in the bones and joints through potential binding to bone marrow fibroblasts. In the context of our findings, this could indicate that both defluorination and radioligand accumulation in the bones and joints are occurring and therefore, it is non-trivial to determine the extent of the fluorosulfate decomposition using the FAPI derivative biovector using PET imaging experiments.

## 5. Conclusions

In conclusion, the rapid preparation of two ^18^F-labeled tracers targeting FAP was achieved by harnessing the ultra-fast reaction kinetics of the [^18^F]SuFEx reaction. Furthermore, the purification of the final radiofluorinated products with SPE, thereby avoiding time-consuming semi-preparative HPLC purification, is a distinct advantage of this protocol compared to contemporary literature preparations and facilitated the implementation of the radiosynthetic method into a commercially available automated radiosynthesizer. As far as the authors are aware, our work presents one of the highest activity yields for a radiofluorinated FAPI reported thus far. Notably, the work sheds light on the stability of the [^18^F]aryl-fluorosulfate motif, with a comparison of our findings with the existing literature being discussed. We believe our findings will aid and accelerate further applications of the advantageous [^18^F]SuFEx chemistry towards PET tracer development.

## Figures and Tables

**Table 1:** IC_50_ values derived from FAPα enzyme inhibition by compounds **12**, **13** and FAPI-04.

Compound	IC_50_ (nM)	Hill Coefficient
12	9.63 (7.55–12.41)	−1.56 (−2.43 to −1.059)
13	4.17 (3.01–5.56)	−1.27 (−1.98 to −0.91)
FAPI-04	6.55 (4.58–9.35)	−1.51 (−2.51 to −0.95)

IC_50_ values and Hill coefficients were obtained by analyzing the sigmoid dose–response curves shown in Figure 1 with nonlinear regression using Equation (2) (see Section 2). IC_50_ values and Hill coefficients are shown as mean values with confidence intervals of 95% given in brackets.

**Table 2:** ^18^F Recovery, ^18^F incorporation (RCC) and activity yield (AY) using different phase transfer additive (PTA) salts.

PTA Salt	^18^F Recovery	Compound	RCC (%) 30 s	RCC (%) 1 min	RCC (%) 5 min	AY (%)
Et_4_NHCO_3_	93 ± 3% (*n* = 4)	[^18^F]**12**	41 ± 35 *	45 ± 33 *	54 ± 30 *	33 ± 23 *
		[^18^F]**13**	27 ± 5 *	33 ± 6 *	48 ± 3 *	28 ± 2 *
BnEt_3_NCl	96 ± 2% (*n* = 6)	[^18^F]**12**	60 ± 13	65 ± 9	71 ± 9	52 ± 2
		[^18^F]**13**	44 ± 2	48 ± 2	61 ± 2	36 ± 1
BnBu_3_NCl	97 ± 1% (*n* = 6)	[^18^F]**12**	62 ± 2	68 ± 1	73 ± 2	55 ± 1
		[^18^F]**13**	44 ± 11	44 ± 12	50 ± 10	43 ± 3

Reaction conditions as described in Section 2. RCC (radiochemical conversion) was analyzed using radio-HPLC. The AY (activity yield) was obtained by dividing the measured activity of the product by the measured starting activity of [^18^F]F^−^ prior to radiosynthesis and multiplying by one hundred. Data shown are mean values (±SD) and, unless noted otherwise, each experiment was performed in triplicate. * Experiments performed in duplicate.

## Data Availability

Data are contained within the article and Supplementary Material.

## Supplementary Materials

The following supporting information can be downloaded at: https://www.mdpi.com/article/10.3390/pharmaceutics15122749/s1, Experimental Procedures for Organic Syntheses, Copies of HPLC and NMR data and FAP assay data are provided within the supporting information. Detailed synthetic procedures and characterization data for the preparation of aryl-fluorosulfate FAPIs; Figures S1–S6: NMR data of aryl-fluorosulfate FAPIs; Figures S7 and S8: HR-MS chromatograms of aryl-fluorosulfate FAPIs; Figures S9 and S10: Analytical HPLC chromatograms of aryl-fluorosulfate FAPIs. Tables S1–S5: Optimization data for preparation of radiolabeled aryl-fluorosulfate FAPIs. Figures S11–S20: Radio-(U)HPLC chromatograms of radiolabeled aryl-fluorosulfate FAPIs. Figures S21 and S22: Calibration curves for the molar activity determination of radiolabeled aryl-fluorosulfate FAPIs. Figure S23: Radioactivity-detected HPLC chromatograms of a radiolabeled aryl-fluorosulfate FAPI after incubation with human and rat liver cytosol. Table S6: Composition of Supersol solubilizing agent. Figure S24: Linearity of fluorescence increase, catalyzed by human recombinant FAPα processing of a peptide substrate.
