# Supplementary material for: Preparation of 18F-Labeled Tracers Targeting Fibroblast Activation Protein via Sulfur [18F]Fluoride Exchange Reaction
Source: Pharmaceutics. 2023 Dec 10;15(12):2749. doi: 10.3390/pharmaceutics15122749 (PMC10747913; doi:10.3390/pharmaceutics15122749)
Supplement: Supplementary file 1 [file pharmaceutics-15-02749-s001.zip › pharmaceutics-2676119-supplementary.pdf]

Supporting Information

Preparation of ^{18}F-Labeled Tracers Targeting Fibroblast Activated Protein via Sulfur [^{18}F]Fluoride Exchange Reaction

Austin Craig,[1] Jürgen Kogler,[1,2] Markus Laube,[1] Martin Ullrich,[1] Cornelius K. Donat,[1] Robert Wodtke,[1] Klaus Kopka,[1,2] Sven Stadlbauer[1*]

[1]Helmholtz-Zentrum Dresden-Rossendorf, Institute of Radiopharmaceutical Cancer Research, Bautzner Landstrasse 400, 01328 Dresden, Germany.

[2]Faculty of Chemistry and Food Chemistry, School of Science, TU Dresden, Dresden, Germany.

Corresponding Author: s.stadlbauer@hzdr.de

Contents

Organic Synthesis 3

General Experimental Section 3

Preparation of Literature Known Compounds 4

Novel Preparation Methods and Synthesis of Novel Compounds 9

^{1}H, ^{13}C and ^{19}F NMR Spectra of Novel Compounds 13

HR-MS of Novel Compounds 16

HPLC Chromatograms of HPLC Purified Compounds 17

Radiochemistry 18

Preparation of ^{18}F-labeled Tracers Targeting Fibroblast Activation Protein 18

Radio-HPLC and Radio-UHPLC Chromatograms 19

Liver Microsome Experiments 24

Composition of Supersol 24

References 25

Organic Synthesis

General Experimental Section

Unless otherwise stated, all commercial reagents (for example, compounds **14**, **15**, **19**, **22**, **24**, **29** and **31**) and solvents were obtained and used without further purification. NMR spectroscopy analysis was performed on an Agilent Technologies 400 MR spectrometer consisting of 400/54 premium compact magnet, 400 MR console, and 400 MHz OneNMRProbe PT probe head (400 MHz for ^{1}H, 101 MHz for ^{13}C, and 376 MHz for ^{19}F). ^{1}H and ^{13}C chemical shifts are reported in ppm relative to the residual solvent shift in methanol-d_4 (δ_H = 3.31; δ_C = 49.0) or chloroform-d_3 (δ_H = 7.26; δ_C = 77.36), and ^{19}F chemical shifts were reported relative to $CFCl_3$ as internal standard (δ_F = 0.00). The observed signal multiplicities are characterized as follows: s = singlet, d = doublet, t = triplet, q = quartet, quin = quintet, m= multiplet, and br = broad. Coupling constants (*J* values) were reported in hertz (Hz). HRMS analysis was performed on a Xevo G2-XS QTof Quadrupole Time-of-Flight system (Waters Corporation Milford, Massachusetts, United States). All NMR spectra were analyzed and processed using the software MestreNova (version 6.1.1-6384). Thin-layer chromatography (TLC) analyses were carried out using Merck silica gel 60 F_{254} aluminium plate sheets and visualized under a UV detector (λ: 254 nm). Compound purification using preparative column chromatography was performed using Merck silica gel (mesh size 230–400 ASTM) with the appropriate mobile phases as specified for the respective compounds. The utilized system is a Shimadzu prominence modular HPLC system (Shimadzu Corporation, Kyoto, Japan) with a DGU-20A 5R degasser, two LC-20AR pumps, a CTO-20AC column oven with column switching valve, a SIL-20AC HT autosampler for analytical samples, a SPD-M20A diode array detector, a FRC-10A fraction collector and a CBM-20A communication bus module. All analytical and preparative samples were separated on the column at 40 °C and detected at 254 nm. For analytical HPLC samples a C-18 Jupiter Proteo (Phenomenex Inc., Torrance, CA, USA; 250 × 4.6 mm, 4 µm, 90 Å) column and the following gradient was used: $t_{0\text{-}5\ min}$ = 05%, $t_{5\text{-}25\ min}$ = 5→ 95%, $t_{25\text{-}31\ min}$ = 95%, $t_{31\text{-}32\ min}$ = 95→ 5%, $t_{32\text{-}42\ min}$ = 5% flow rate = 1 mL/ min; mobile phase: MeCN + 0.1% TFA in water + 0.1% TFA. Preparative HPLC purifications were performed on a C-18 Jupiter Proteo (Phenomenex Inc., Torrance, CA, USA; 250 × 21.2 mm, 4 µm, 90 Å) column.

Compounds **16-18**, **20**, **21**, **23**, **25**, **26**, **27** and **28** were prepared according to or adapted from the previously reported protocols of K. Jansen et al., and T. Lindner et al. [1,2]. The confirmation of the aforementioned compounds using NMR were found to be consistent with the spectral shifts as previously described.

Preparation of Literature Known Compounds

tert-Butyl (*S*)-(2-(2-carbamoyl-4,4-difluoropyrrolidin-1-yl)-2-oxoethyl)carbamate (16)

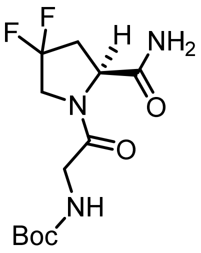

16

A solution of *N*-Boc-L-glycine (**15**, 5.556 g, 31.71 mmol, 1.2 eq.), HATU (19.78 g, 52.02 mmol, 2.0 eq.) and DIPEA (1.0 mL, 63.15 mmol, 2.5 eq.) in DMF (40 mL) was stirred for 30 minutes at 20 °C. A solution of 4,4-Diflouro-L-prolinamide hydrochloride (**14**, 4.836 g, 25.92 mmol, 1.0 eq.) and DIPEA (11 mL, 63.15 mmol, 2.5 eq.) in a mixture of DMF and DCM (1:1, 40 mL) was added and the reaction was stirred for 14 hours at 23 °C. The resulting white precipitate was filtered off, washed with H_2O and cold DCM. The crude product was recrystallized from EtOAc. The product was obtained as white solid (3.236 g, 10.530 mmol, 41%).

^{1}H NMR (400 MHz, DMSO-d_6) δ 7.74 – 7.08 (m, 2H), 6.92 – 6.81 (m, 1H), 4.45 (dd, *J* = 9.7, 4.2 Hz, 1H), 4.14 – 4.02 (m, 1H), 4.01 – 3.87 (m, 1H), 3.85 – 3.64 (m, 1H), 2.81 – 2.63 (m, 1H), 2.42 – 2.27 (m, 1H), 1.38 (s, 9H).

tert-Butyl (*S*)-(2-(2-cyano-4,4-difluoropyrrolidin-1-yl)-2-oxoethyl)carbamate (17)

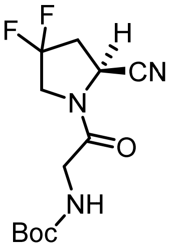

17

A Schlenk round-bottom flask with compound **16** (3.000 g, 8.762 mmol, 1.0 eq.) was evacuated and filled with argon three times. THF (40 mL, anhydrous) and DIPEA (10 mL, 57.408 mmol, 6 eq.) was added. A solution of TFAA (5.0 mL, 35.971 mmol, 3.5 eq.) in DCM (5.0 mL, anhydrous) was added over 10 minutes via an addition funnel, while cooling to 0 °C. The reaction mixture was allowed to reach room temperature and stirred for 3 hours. The reaction mixture was washed with 1 M aqueous hydrochloric acid solution, saturated sodium bicarbonate solution and brine. The organic phase was dried over sodium sulfate, filtered and the solvent evaporated. The crude product was purified by column chromatography on silica

(ethyl acetate/cyclohexane: 2/3). The product was obtained as yellow solid (1.566 g, 5.379 mmol, 55%).

^{1}H NMR (400 MHz, $CDCl_3$) δ 5.42 (s, 1H), 4.96 (t, *J* = 6.5 Hz, 1H), 4.04 – 3.78 (m, 4H), 2.82 – 2.68 (m, 2H), 1.44 (s, 9H).

(*S*)-4,4-Difluoro-1-glycylpyrrolidine-2-carbonitrile (18)

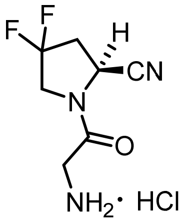

18

Compound **17** (0.886 g, 3.063 mmol) was dissolved in acetonitrile (anhydrous,10 mL) and cooled to 0 °C. 4 M Hydrogen chloride solution in dioxane (5.0 mL) was added and the reaction was stirred at 20 °C for 90 minutes. The solvent was evaporated under reduced pressure, and the product was obtained as off-white solid (0.691 g, 3.063 mmol, quantitative yield).

^{1}H NMR (400 MHz, D_2O): (5/1 mixture of *trans*/*cis* amide rotamers) δ 5.39 (dd, *J* = 8.9, 2.6 Hz, 0.1 H), 5.23 – 5.17 (m, 0.9 H), 4.28 – 4.05 (m, 2H), 4.03 (s, 2H), 3.03 – 2.92 (m, 2H).

Methyl 6-methoxyquinoline-4-carboxylate (20)

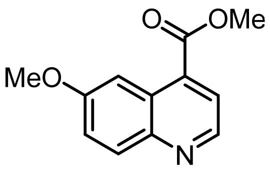

20

6-Methoxyquinoline-4-methyl carboxylic acid (**19**, 3.675 g, 18.086 mmol, 1 eq.) was suspended in MeOH (60 mL). Concentrated H_2SO_4 (5.0 mL, 93.301 mmol, 5 eq.) was added while cooling to 0 °C. The reaction was stirred at 70 °C for 18 hours. The product was precipitated from the solution by addition of 5 M aqueous sodium hydroxide solution. The resulting precipitate was washed with saturated sodium bicarbonate solution and H_2O. After drying the product was obtained as off-white solid (3.620 g, 16.665 mmol, 92%).

^{1}H NMR (400 MHz, $CDCl_3$) δ 8.84 (d, *J* = 4.5 Hz, 1H), 8.23 (d, *J* = 2.8 Hz, 1H), 8.04 (d, *J* = 9.2 Hz, 1H), 7.91 (d, *J* = 4.5 Hz, 1H), 7.41 (dd, *J* = 9.2, 2.8 Hz, 1H), 4.02 (s, 3H), 3.96 (s, 3H).

Methyl 6-hydroxyquinoline-4-carboxylate (21)

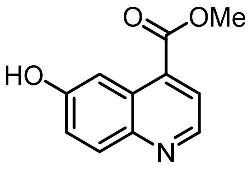

21

Compound **20** (3.600 g, 16.573 mmol, 1 eq.) was dissolved in DCM (anhydrous, 30 mL) under argon atmosphere and cooled to 0 °C. BBr_3 solution (1 M in DCM, 45 mL, 41.43 mmol, 2.5 eq.) was added via an addition funnel over 15 min. The reaction was allowed to warm to 20 °C and stirred for 18 hours. Sodium bicarbonate solution was added and the aqueous phase was extracted with ethyl acetate. The combined organic layers were washed with saturated sodium chloride solution, dried over Na_2SO_4, filtered and the solvent evaporated under reduced pressure. The product was obtained as yellow solid. (2.740 g, 13.484 mmol, 81%).

^{1}H NMR (400 MHz, DMSO-*d*$_6$) δ 10.48 (s, 1H), 8.87 (d, *J* = 4.6 Hz, 1H), 8.02 (d, *J* = 9.1 Hz, 1H), 7.98 (d, *J* = 2.7 Hz, 1H), 7.95 (d, *J* = 4.6 Hz, 1H), 7.46 (dd, *J* = 9.1, 2.7 Hz, 1H), 3.98 (s, 3H).

Methyl 6-(4-chloropropoxy)quinoline-4-carboxylate (23)

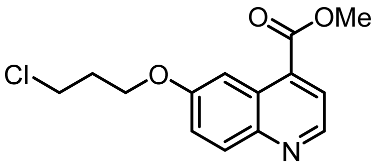

23

1-Bromo-3-chloropropane (**22**, 1.833 mL, 11.642 mmol, 1.1 eq.) was added to a mixture of compound **21** (2.142 g, 10.541 mmol, 1 eq.) and Cs_2CO_3 (6.949 g, 21.328 mmol, 2 eq.) in DMF (100 mL). The reaction was stirred at 20 °C for 16 hours. The solvent was evaporated under reduced pressure. The residue was partitioned between DCM and saturated sodium bicarbonate solution. The layers were separated and the aqueous phase extracted with DCM. The combined organic layers were washed with brine, dried over Na_2SO_4, filtered and the solvent was evaporated. The crude product was purified using column chromatography on silica. The product was obtained as yellow solid (1.826 g, 6.528 mmol, 62%).

^{1}H NMR (400 MHz, $CDCl_3$) δ 8.82 (d, *J* = 4.5 Hz, 1H), 8.22 (d, *J* = 2.8 Hz, 1H), 8.03 (d, *J* = 9.2 Hz, 1H), 7.88 (d, *J* = 4.5 Hz, 1H), 7.38 (dd, *J* = 9.2, 2.8 Hz, 1H), 4.27 (t, *J* = 5.8 Hz, 2H), 4.00 (s, 3H), 3.77 (t, *J* = 6.3 Hz, 2H), 2.30 (p, *J* = 6.1 Hz, 2H).

Methyl 6-(3-(4-(tert-butoxycarbonyl)piperazin-1-yl)propoxy)quinoline-4-carboxylate (25)

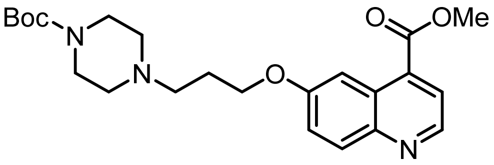

25

1-Boc-piperazine (**24**, 5.495 g, 25.502 mmol, 5 eq.) and KI (2.745 g, 16.536 mmol, 3 eq.) were added to a solution of compound **23** (1.348 g, 4.819 mmol, 1 eq.) and DIPEA (12.59 mL, 72.285 mmol, 15 eq.) in DMF (25 mL). The reaction was stirred at 60 °C for 18 hours. The solvent was evaporated under reduced pressure. The residue was partitioned between ethyl acetate and saturated sodium bicarbonate solution. The layers were separated and the aqueous phase was extracted with ethyl acetate. The combined organic phases were washed with brine, dried over Na_2SO_4, filtered and the solvent was removed under reduced pressure. The crude product was purified using column chromatography on silica gel. The product was obtained as yellow solid (1.124 g, 2.617 mmol, 54%).

^{1}H NMR (400 MHz, $CDCl_3$) δ 8.83 (d, *J* = 4.5 Hz, 1H), 8.22 (d, *J* = 2.8 Hz, 1H), 8.03 (d, *J* = 9.2 Hz, 1H), 7.90 (d, *J* = 4.5 Hz, 1H), 7.39 (dd, *J* = 9.2, 2.8 Hz, 1H), 4.19 (t, *J* = 6.2 Hz, 2H), 4.01 (s, 3H), 3.45 (t, *J* = 5.0 Hz, 4H), 2.58 (t, *J* = 7.3 Hz, 2H), 2.43 (t, *J* = 5.1 Hz, 4H), 2.06 (dt, *J* = 7.9, 6.4 Hz, 2H), 1.45 (s, 9H).

6-(3-(4-(tert-Butoxycarbonyl)piperazin-1-yl)propoxy)quinoline-4-carboxylic acid (26)

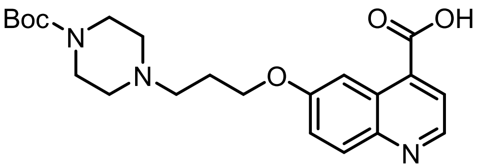

26

A solution of LiOH (0.123 g, 5.136 mmol, 5 eq.) in H_2O (5 mL) was added to a solution of compound **4** (0.396 g, 0.922 mmol, 1 eq.) and the reaction was stirred at 23 °C for 16 hours. The solvent was removed under reduced pressure. The crude was purified by column chromatography on silica (DCM/MeOH, 8:2 +1% TEA). The product was dissolved in acetonitrile and filtered to remove silica particles. The product was obtained as off-white solid (89%, 0.341 g, 0.821 mmol).

^{1}H NMR (400 MHz, CD_3OD) δ 8.62 (d, *J* = 4.5 Hz, 1H), 7.91 – 7.86 (m, 1H), 7.58 (d, *J* = 4.5 Hz, 1H), 7.34 (dd, *J* = 9.2, 2.7 Hz, 1H), 4.16 (t, *J* = 6.1 Hz, 2H), 3.46 (t, *J* = 5.1 Hz, 4H), 2.69 – 2.61 (m, 2H), 2.52 (t, *J* = 5.1 Hz, 4H), 2.13 – 2.00 (m, 2H), 1.45 (s, 9H).

tert-Butyl(*S*)-4-(3-((4-((2-(2-cyano-4,4-difluoropyrrolidin-1-yl)-2-oxoethyl)carbamoyl)quinolin-6-yl)oxy)propyl)piperazine-1-carboxylate (27)

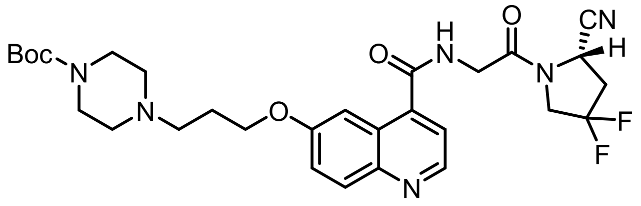

27

Compound **26** (0.282 g, 0.679 mmol, 1.0 eq.), HATU (0.959 g, 2.522 mmol, 3.0 eq.), and DIPEA (0.250 mL, 1.435 mmol, 2.5 eq.) were dissolved in DMF (5.0 mL). The solution was stirred at 23 °C for 20 minutes. A solution of compound **18** (0.151 g, 0.669 mmol, 1.0 eq.) and DIPEA (0.250 mL, 1.435 mmol, 2.5 eq.) was added and the reaction was stirred at 23 °C for 18 hours. The solvent was removed under reduced pressure. The residue was dissolved in ethyl acetate and washed with saturated bicarbonate solution and brine. The organic phase was dried over Na_2SO_4, filtered and the solvent was evaporated under reduced pressure. The crude was purified by column chromatography on silica (DCM/MeOH, 25→15:1 + 0.5% TEA). The product was obtained as brownish solid (0.185 g, 0.315 mmol, 47%).

^{1}H NMR (400 MHz, CD_3OD) δ 8.74 (d, *J* = 4.5 Hz, 1H), 7.97 (d, *J* = 9.3 Hz, 1H), 7.93 (d, *J* = 2.8 Hz, 1H), 7.56 (d, *J* = 4.4 Hz, 1H), 7.46 (dd, *J* = 9.2, 2.8 Hz, 1H), 5.13 (dd, *J* = 9.3, 3.2 Hz, 1H), 4.37 – 3.94 (m, 7H), 3.48 – 3.39 (m, 4H), 3.01 – 2.67 (m, 2H), 2.63 (dd, *J* = 8.6, 6.6 Hz, 2H), 2.47 (t, *J* = 5.1 Hz, 4H), 2.13 – 2.04 (m, 2H), 1.46 (s, 9H).

(*S*)-N-(2-(2-cyano-4,4-difluoropyrrolidin-1-yl)-2-oxoethyl)-6-(3-(piperazin-1-yl)propoxy)quinoline-4-carboxamide (28)

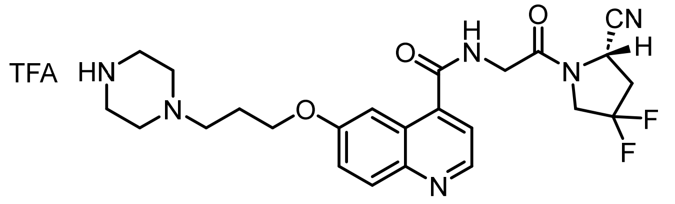

28

Compound **27** (0.185 g, 0.315 mmol) was dissolved in DCM (1.0 mL) and cooled to 0 °C. TFA (1.0 mL) was added and the reaction stirred for 1 hour at 23 °C. The solvent was removed under reduced pressure. The crude product was purified by preparative HPLC using the following gradient: $t_{0\text{-}5\ min}$ = 5% MeCN, $t_{5\text{-}25\ min}$ = 5→15%, $t_{25\text{-}26\ min}$ = 15→95%, $t_{26\text{-}31\ min}$ = 95%, $t_{31\text{-}32\ min}$ = 95→05%, $t_{32\text{-}42\ min}$ = 5%).

HPLC (gradient 2): t_R = 16.14 min

^{1}H NMR (400 MHz, CD_3OD) δ 8.82 (d, *J* = 4.8 Hz, 1H), 8.06 (d, *J* = 2.7 Hz, 1H), 8.01 (d, *J* = 9.3 Hz, 1H), 7.67 (d, *J* = 4.8 Hz, 1H), 7.54 (dd, *J* = 9.3, 2.7 Hz, 1H), 5.10 (dd, *J* = 9.4, 3.3 Hz, 1H), 4.36 (t, *J* = 6.0 Hz, 2H), 4.29 (d, *J* = 1.8 Hz, 2H), 4.27 – 4.17 (m, 1H), 4.16 – 4.03 (m, 1H), 3.43 (t, *J* = 5.2 Hz, 4H), 3.27 (p, *J* = 1.6 Hz, 4H), 3.20 (t, 7.8 Hz 2H), 2.99 – 2.84 (m, 1H), 2.84 – 2.71 (m, 1H), 2.33 – 2.19 (m, 2H).

Novel Preparation Methods and Synthesis of Novel Compounds

4-((Fluorosulfonyl)oxy)benzoic acid (30)

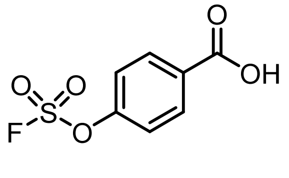

30

4-Hydroxybenzoic acid (**29**, 0.345 g, 2.498 mmol, 1.0 eq.) and 4-(acetylamino)phenyl]imidodisulfuryl difluoride (AISF) (0.292 g, 0.929 mmol, 1.8 eq.) were dissolved in THF (3.0 mL). DBU (1.15 mL, 7.732 mmol, 1.8 eq.) was added dropwise. The reaction was stirred for 15 minutes at 23 °C. The solvent was removed under reduced pressure. The residue was dissolved in ethyl acetate, washed with aqueous 0.5 M HCl solution and brine, dried over Na_2SO_4, filtered and the solvent removed under reduced pressure. The crude product was purified by column chromatography on silica (ethyl acetate/ *n*-hexane, 1:3

+ 0.1% formic acid). The product was obtained as white powder (0.068 g, 0.309 mmol, 12%). The analytical data collected are in accordance with the literature [3].

(*S*)-4-(4-(3-((4-((2-(2-cyano-4,4-difluoropyrrolidin-1-yl)-2-oxoethyl)carbamoyl)quinolin-6-yl)oxy)propyl)piperazine-1-carbonyl)phenyl sulfurofluoridate (12)

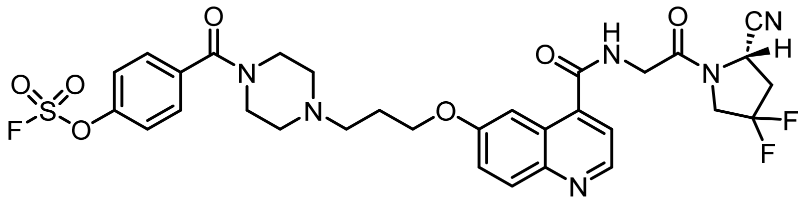

12

Compound **28** (8.90 mg, 40.4 µmol, 1.8 eq.), DIPEA (12.0 µL, 69.4 µmol, 1.5 eq.) and HATU (25.6 mg, 67.3 µmol, 3.0 eq.) were dissolved in DMF (250 µL) and stirred for 10 minutes at 23 °C. A solution of compound **18** (11.0 mg, 22.6 µmol, 1.0 eq.) in DMF (500 µL) was added and the reaction was stirred at 23 °C for 1 hour. The solvent was removed under reduced pressure. The crude product was purified by preparative HPLC using the following gradient:

$t_{0\text{-}5\ min}$ = 30%, $t_{5\text{-}25\ min}$ = 30→ 80%, $t_{25\text{-}26\ min}$ = 80→ 95% $t_{26\text{-}31\ min}$ = 95%, $t_{31\text{-}32\ min}$ = 95→ 30%, $t_{32\text{-}42\ min}$ = 30%; flow rate = 10 mL/min; mobile phase: MeCN + 0.1% TFA in water + 0.1% TFA. The product was obtained as fluffy white solid after lyophilization (11.6 mg, 16.84 µmol, 47%).

Analytical HPLC t_R: 20.45 min

^{1}H NMR (400 MHz, CD_3OD) δ 8.94 (d, *J* = 5.0 Hz, 1H), 8.22 (d, *J* = 2.7 Hz, 1H), 8.11 (d, *J* = 9.3 Hz, 1H), 7.84 (d, *J* = 5.0 Hz, 1H), 7.73 – 7.66 (m, 3H), 7.60 (d, *J* = 8.5 Hz, 2H), 5.13 (dd, *J* = 9.4, 3.3 Hz, 1H), 4.46 (t, *J* = 5.9 Hz, 2H), 4.43 – 4.07 (m, 4H), 3.99 – 3.02 (br m, 8H) 3.53 – 3.45 (m, 2H), 3.05 – 2.74 (m, 2H), 2.40 (dq, *J* = 11.7, 6.1 Hz, 2H).

^{13}C NMR (101 MHz, CD_3OD) δ 170.56, 169.51, 168.62, 160.45, 152.53, 149.21, 144.60, 138.72, 136.28, 131.05, 128.78, 128.66, 127.41 (dd, *J* = 247.6, 3.9 Hz), 125.66, 122.77, 120.84, 118.56, 106.47, 67.26, 55.54, 52.85 (t, *J* = 31.1 Hz), 52.73, 45.97 (d, *J* = 5.6 Hz), 43.01, 37.93 (t, *J* = 25.2 Hz), 24.81.

^{19}F NMR (376 MHz, CD_3OD) δ 37.80, -96.93 – -97.81 (m), -103.71 – -104.48 (m).

HRMS: calculated: m/z = 689.2000 $[M+H]^+$, found: m/z = 689.1992 $[M+H]^+$, calculated: m/z = 711.1819 $[M+Na]^+$, found: m/z = 711.1812 $[M+Na]^+$, calculated: m/z = 727.1559 $[M+K]^+$, found: m/z = 727.1552 $[M+K]^+$

3-((Fluorosulfonyl)oxy)benzoic acid (32)

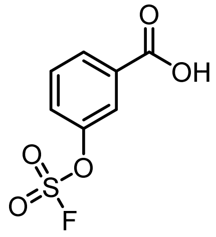

32

3-Hydroxybenzoic acid (**31**, 0.325 g, 2.353 mmol, 1.0 eq.) and 4-(acetylamino)phenyl]imidodisulfuryl difluoride (AISF) (1.334 g, 4.245 mmol, 1.8 eq.) were dissolved in THF (3.0 mL). DBU (1.05 mL, 7.059 mmol, 3.0 eq.) was added dropwise. The reaction was stirred for 15 minutes at 23 °C. The solvent was removed under reduced pressure. The residue was dissolved in ethyl acetate, and washed with aqueous 0.5 M HCl solution and brine. Thereafter, the organic phase was dried over Na_2SO_4, filtered and the solvent was removed under reduced pressure. The crude product was purified by column chromatography on silica (ethyl acetate/cyclohexane, 1:5 + 0.1% formic acid). The product was obtained as white powder (0.046 g, 0.212 mmol, 9%). The analytical data collected are in accordance with the literature [3].

(S)-3-(4-(3-((4-((2-(2-cyano-4,4-difluoropyrrolidin-1-yl)-2-oxoethyl)carbamoyl)quinolin-6-yl)oxy)propyl)piperazine-1-carbonyl)phenyl sulfurofluoridate (13)

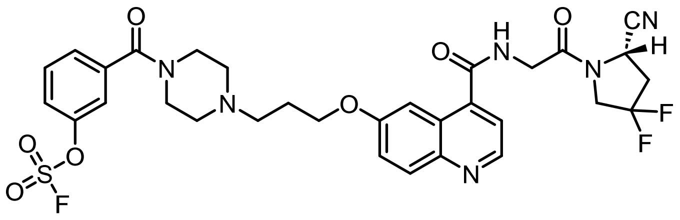

13

Compound **31** (10.50 mg, 47.7 µmol, 2.5 eq.), DIPEA (20.0 µL, 67.2 µmol, 1.5 eq.) and HATU (33.0 mg, 86.79 µmol, 4.4 eq.) were dissolved in DMF (250 µL) and stirred for 10 minutes at 23 °C. A solution of compound **8** (9.6 mg, 19.73 µmol, 1.0 eq.) in DMF (500 µL) was added and the reaction was stirred at 23 °C for 1 hour. The solvent was removed under reduced pressure. The crude product was purified by preparative HPLC using the following gradient:

$t_{0\text{-}5\ min}$ = 30%, $t_{5\text{-}25\ min}$ = 30→ 80%, $t_{25\text{-}26\ min}$ = 80→ 95% $t_{26\text{-}31\ min}$ = 95%, $t_{31\text{-}32\ min}$ = 95→ 30%, $t_{32\text{-}42\ min}$ = 30%; flow rate = 10 mL/min; mobile phase: MeCN + 0.1% TFA in water + 0.1% TFA

The product was obtained as fluffy white solid after lyophilization (11.6 mg, 16.84 µmol, 47%).

Analytical HPLC: t_R = 20.30 min

^1H NMR (400 MHz, CD_3OD) δ 8.98 (d, *J* = 5.1 Hz, 1H), 8.27 (d, *J* = 2.7 Hz, 1H), 8.14 (d, *J* = 9.3 Hz, 1H), 7.91 (d, *J* = 5.1 Hz, 1H), 7.78 – 7.60 (m, 3H), 5.14 (dd, *J* = 9.4, 3.3 Hz, 1H), 4.47 (t, *J* = 5.9 Hz, 2H), 4.44 – 4.05 (m, 4H), 4.05 – 3.02 (br m, 8H), 3.52 – 3.45 (m, 2H), 3.03 – 2.74 (m, 2H), 2.41 (dq, *J* = 12.1, 6.2 Hz, 2H).

^{13}C NMR (101 MHz, CD_3OD) δ 169.91, 169.54, 168.92, 160.23, 151.42, 148.41, 145.14, 139.65, 138.09, 132.53, 128.89, 128.59, 128.12, 127.41 (dd, *J* = 247.6, 3.4 Hz), 126.39, 124.28, 121.46, 120.77, 118.56, 106.39, 67.17, 55.57, 52.91 (t, *J* = 32.0 Hz), 45.99 (d, *J* = 6.0 Hz), 43.00, 37.80 (t, *J* = 25.5 Hz), 24.83.

^{19}F NMR (376 MHz, CD_3OD) δ 36.14 (s), -98.55 – -99.53 (m), -105.31 – -106.17 (m).

HRMS: calculated: m/z = 689.2000 $[M+H]^+$, found: m/z = 689.1993 $[M+H]^+$, calculated: m/z = 711.1819 $[M+Na]^+$, found: m/z = 711.1813 $[M+Na]^+$,calculated: m/z = 727.1559 $[M+K]^+$, found: m/z = 727.1551 $[M+K]^+$

^{1}H, ^{13}C and ^{19}F NMR Spectra of Novel Compounds

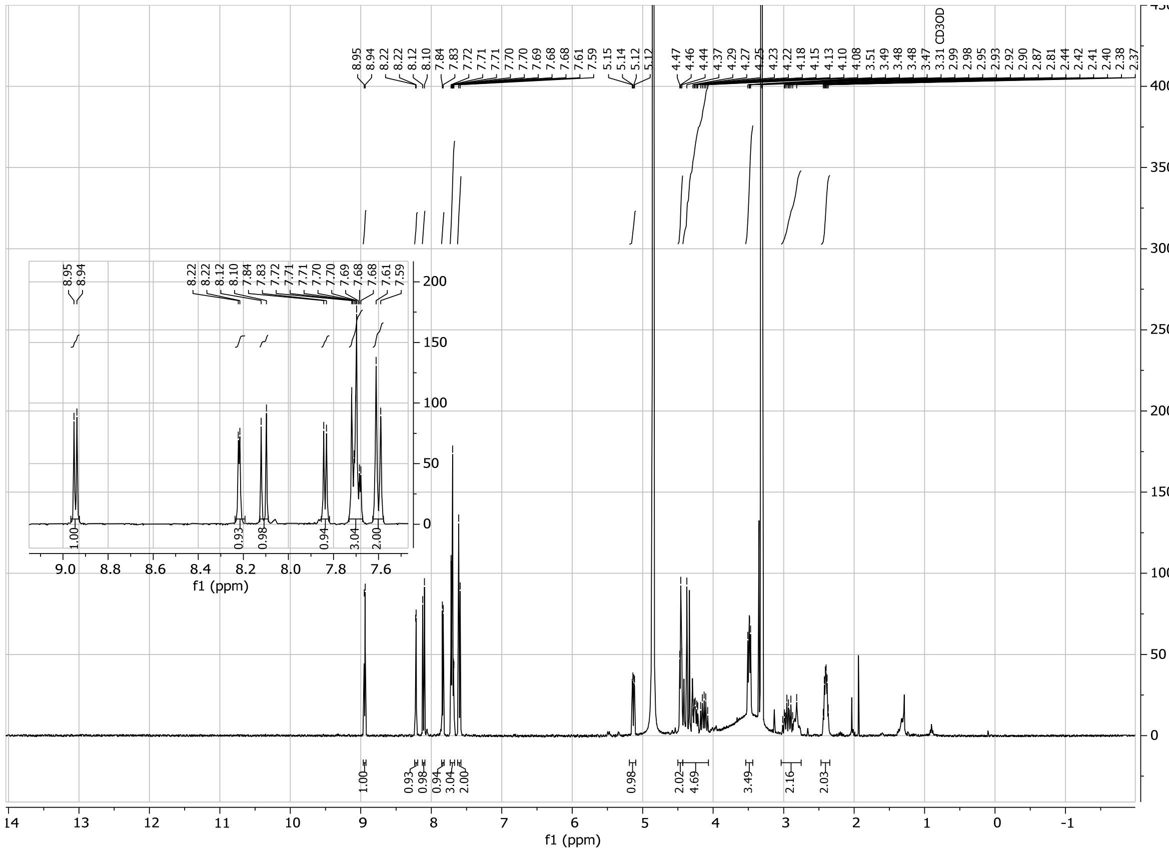

Figure S1. ^{1}H NMR spectrum of **12**.

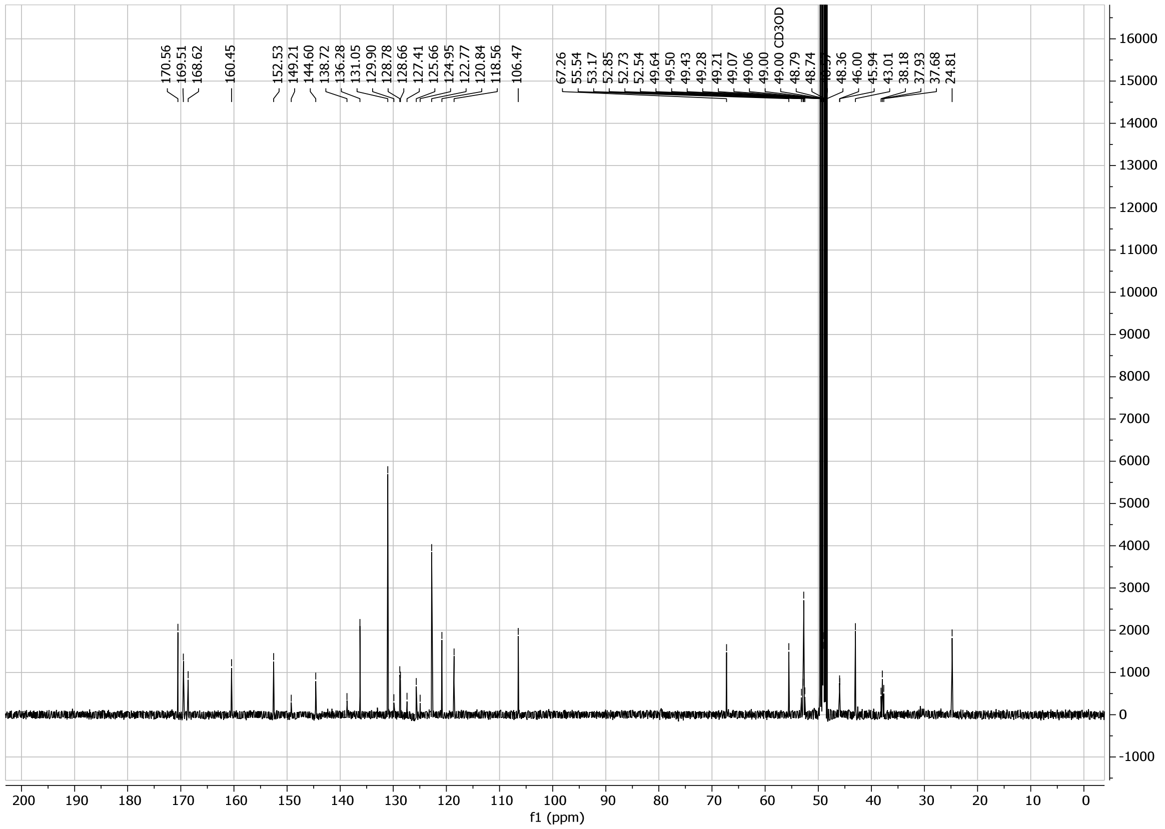

Figure S2. ^{13}C NMR spectrum of **12**.

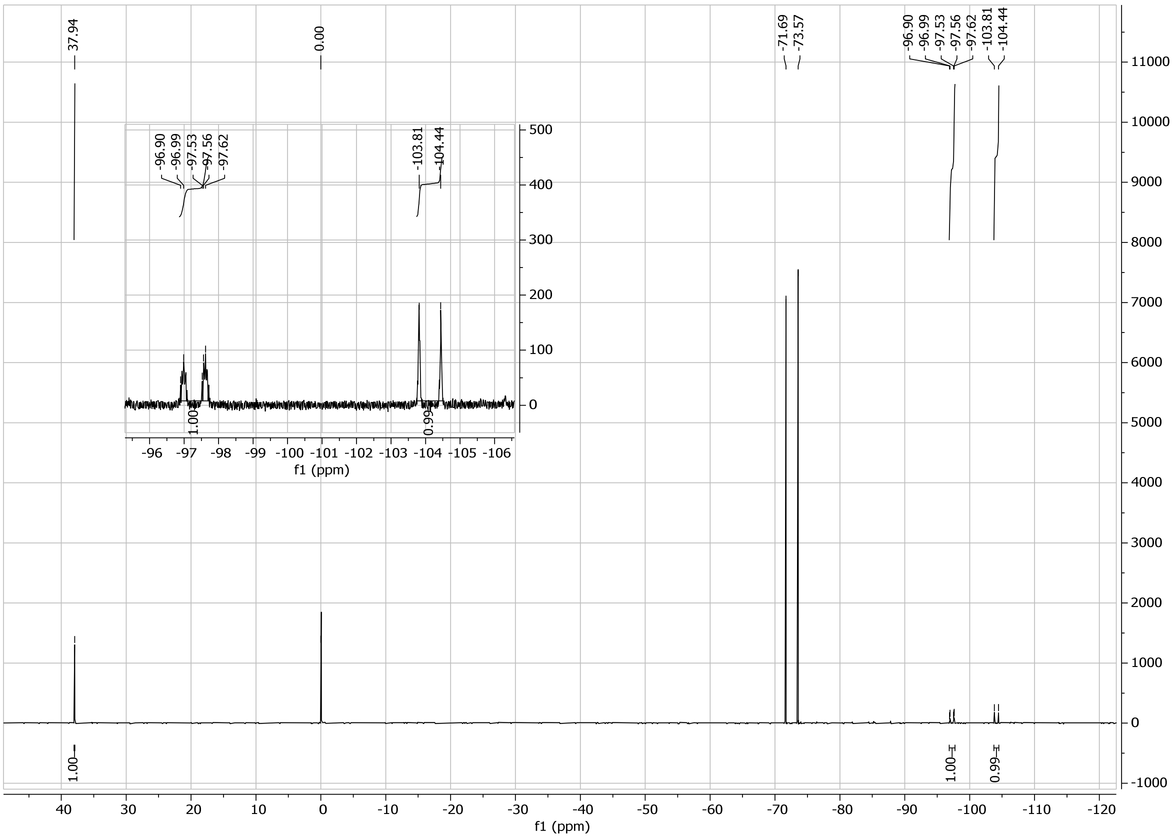

Figure S3. ^{19}F NMR spectrum of **12**.

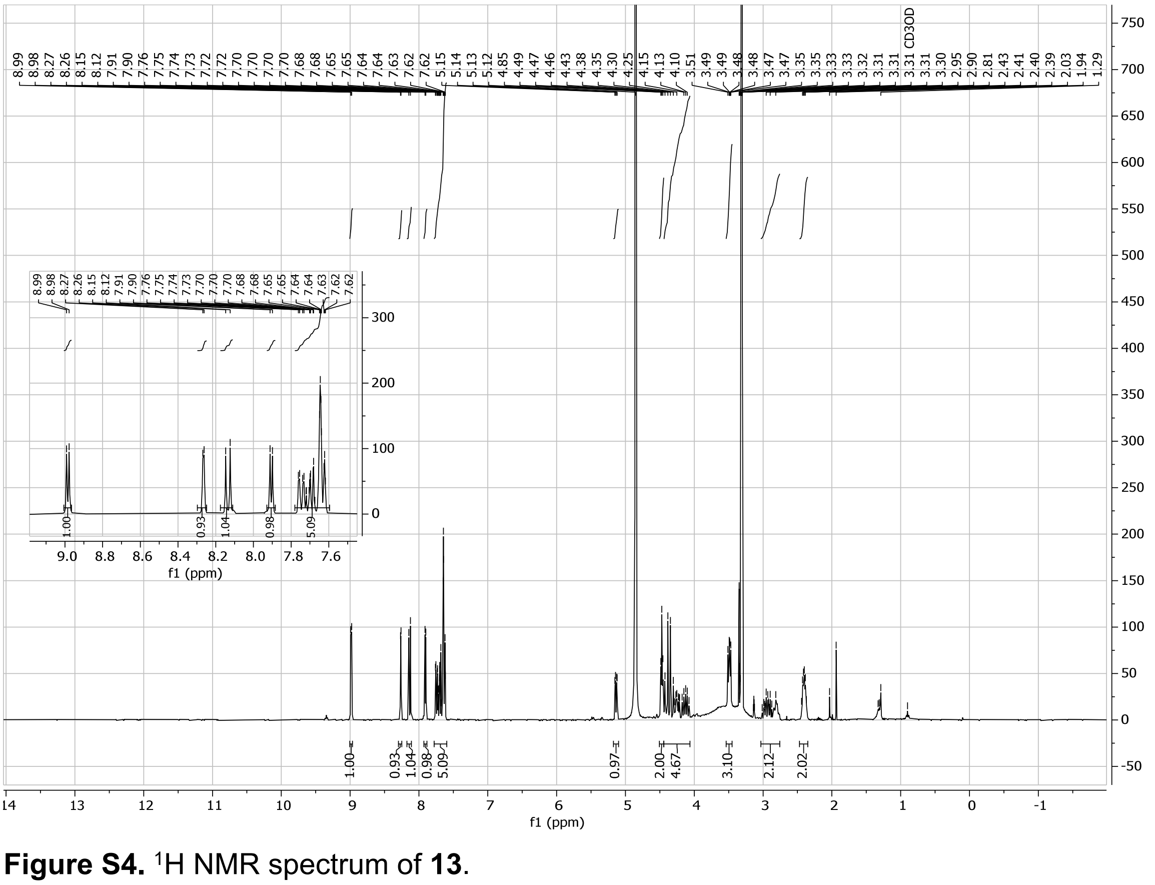

Figure S4. ^{1}H NMR spectrum of **13**.

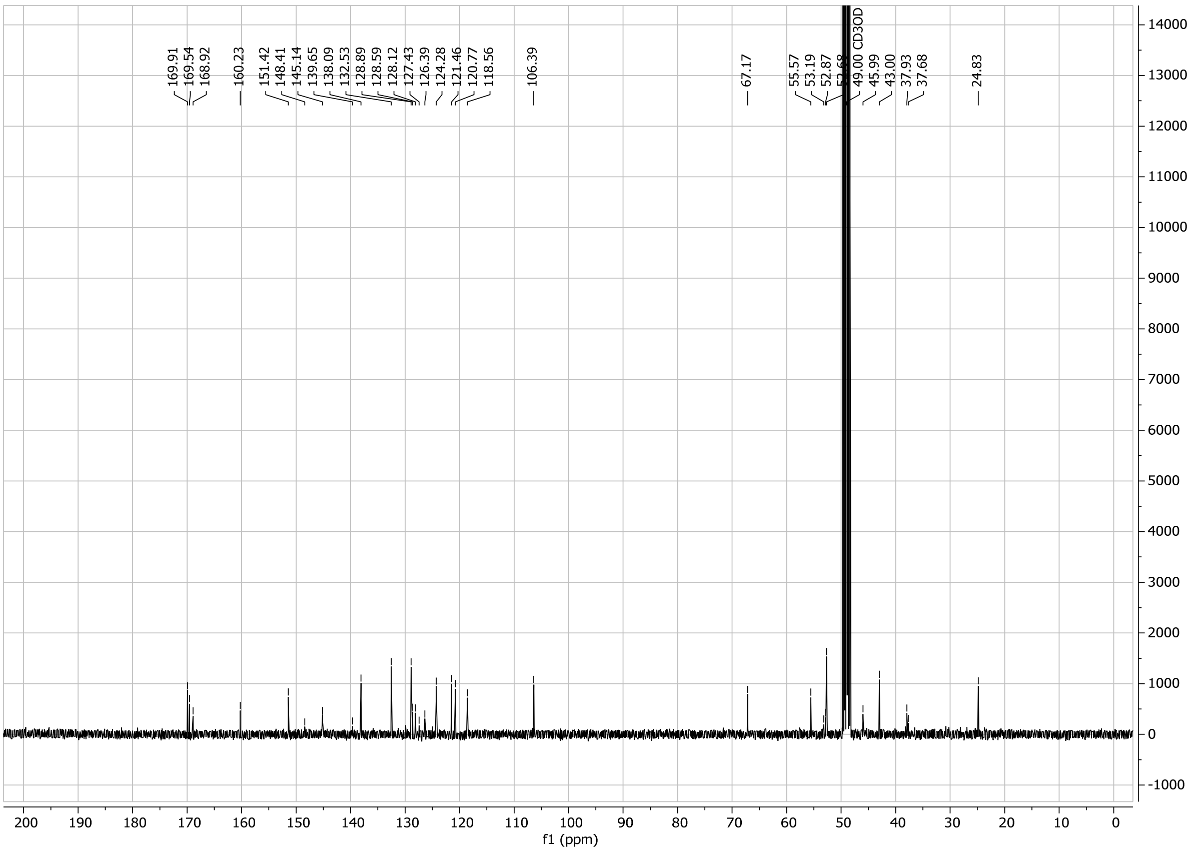

Figure S5. ^{13}C NMR spectrum of **13**.

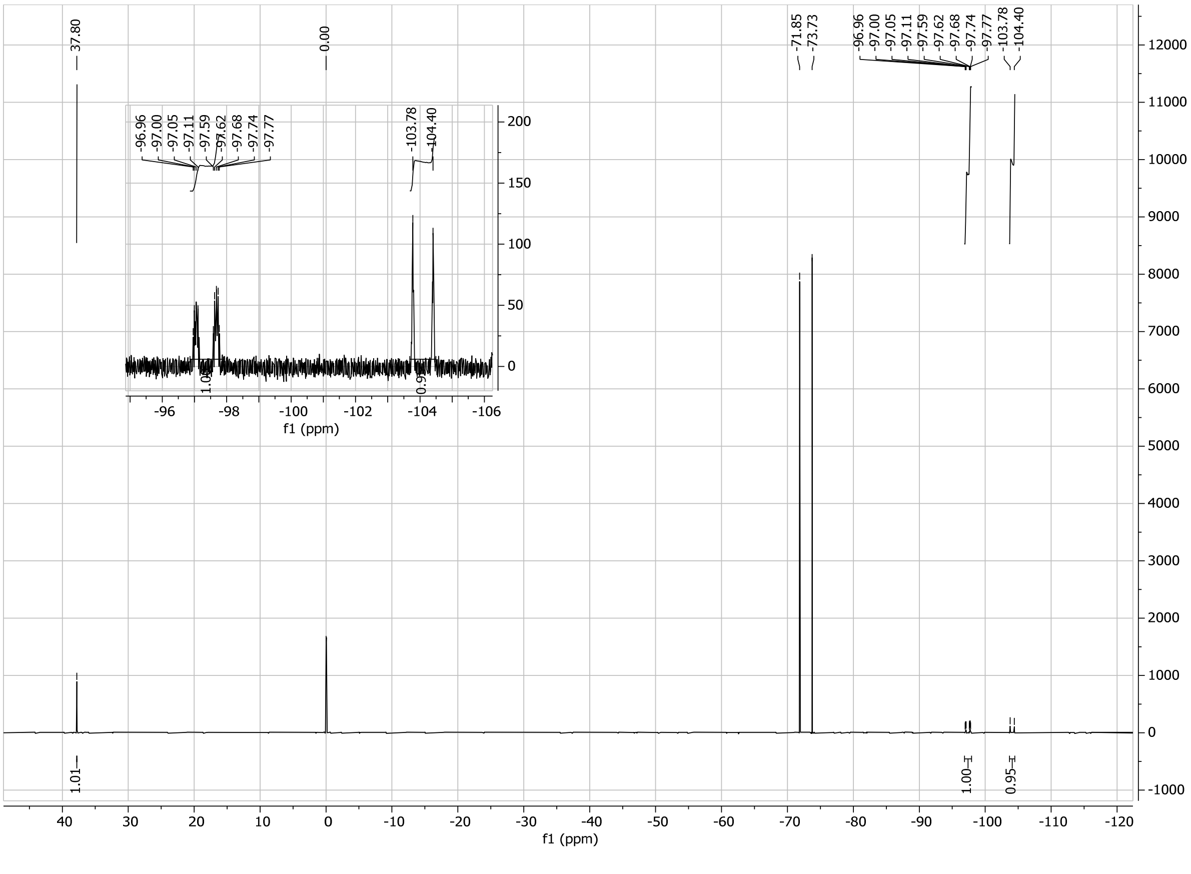

Figure S6. ^{19}F NMR spectrum of **13**.

HR-MS of Novel Compounds

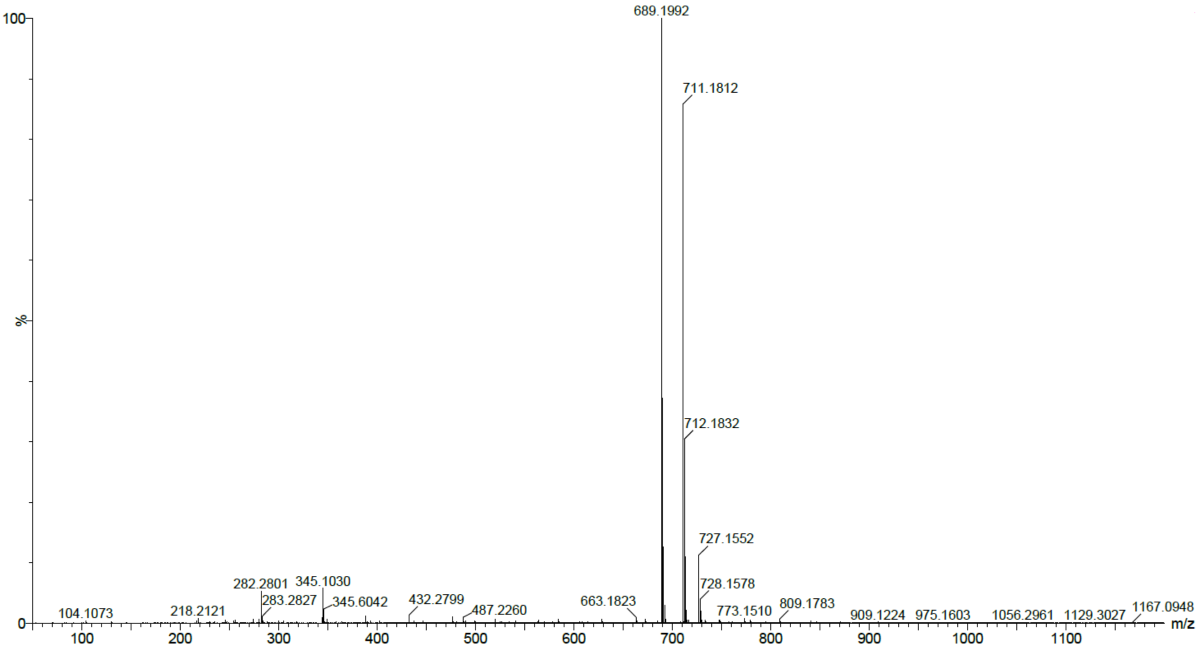

Figure S7. High resolution mass spectrometry chromatogram of **12.**

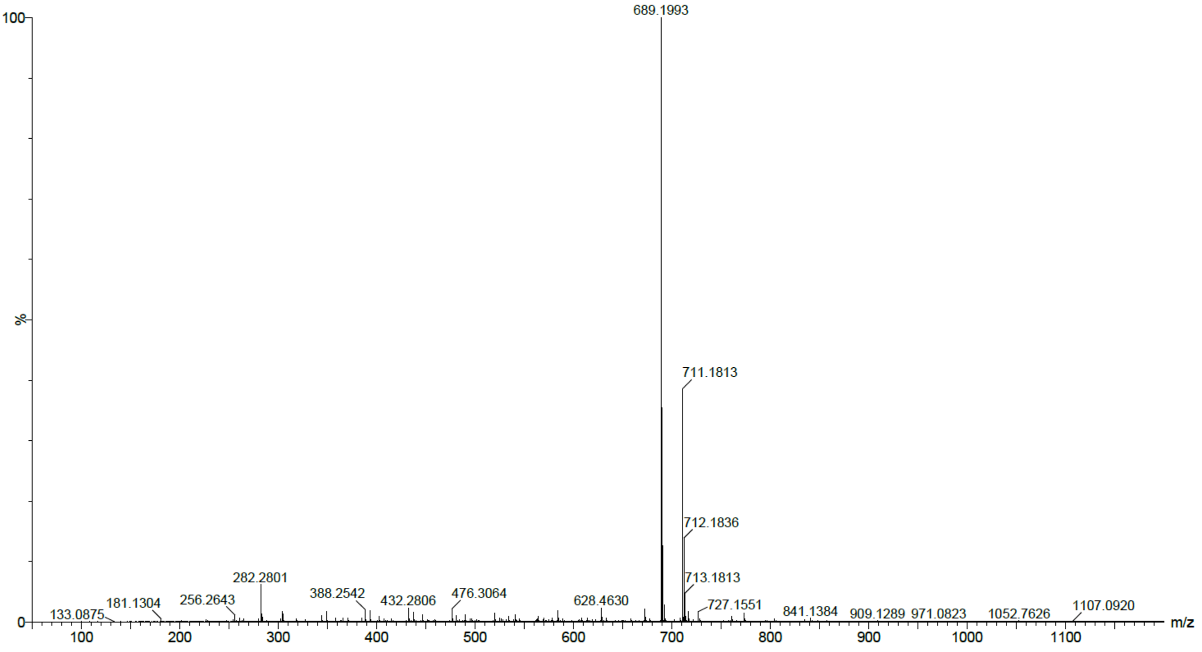

Figure S8. High resolution mass spectrometry chromatogram of compound **13**.

HPLC Chromatograms of HPLC Purified Compounds

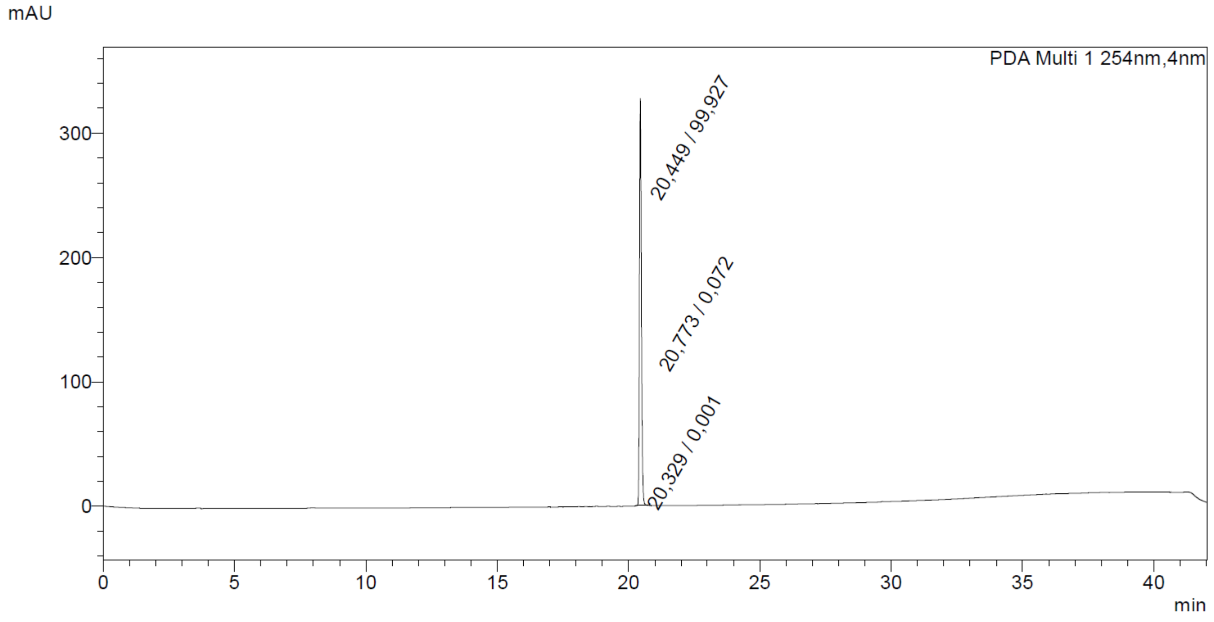

Figure S9. Analytical HPLC chromatogram of **12** (detection at 254 nm).

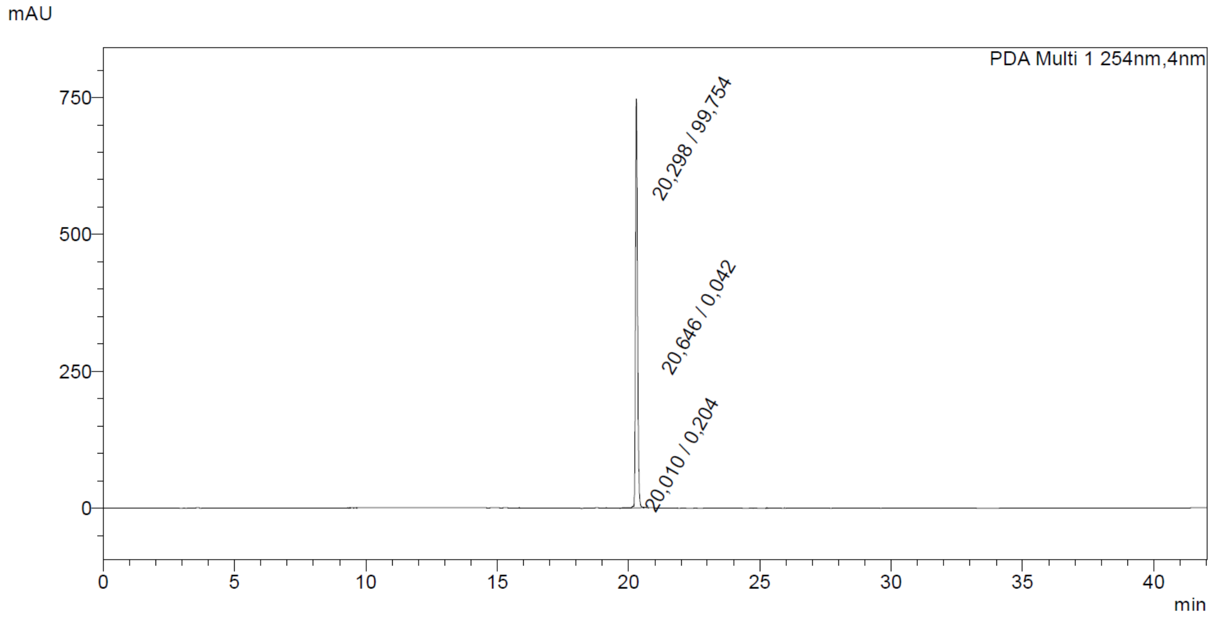

Figure S10. Analytical HPLC chromatogram of **13** (detection at 254 nm).

Radiochemistry

Preparation of ^{18}F-labeled Tracers Targeting Fibroblast Activation Protein

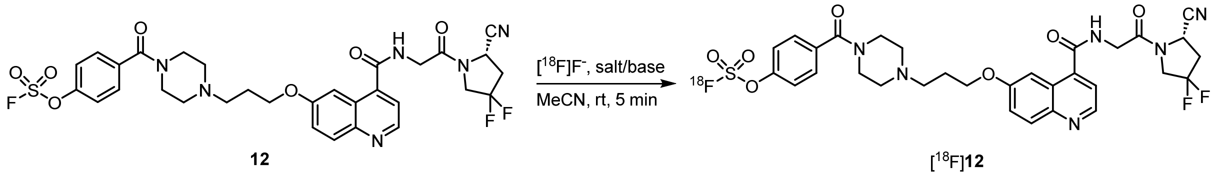

Salt	Run	^{18}F-Elution (%)	RCC (%) 30 sec	RCC (%) 1 min	RCC (%) 5 min	Activity Yield (AY)
$BnEt_3NCl$	**1**	97	45	56	61	54
	**2**	97	70	74	77	52
	**3**	97	64	66	76	51
Et_4NHCO_3	**4**	95	65	68	75	49
	**5**	95	16	21	33	17
$BnBu_3NCl$	**6**	97	62	67	71	54
	**7**	95	60	69	74	54
	**8**	98	63	68	74	56

Table S1. ^{18}F-Elution, radiochemical conversions (RCC) and activity yields of [^{18}F]**12** using different salts.

Salt	^{18}F-Elution (%)	RCC (%) 30 sec	RCC (%) 1 min	RCC (%) 5 min	Activity Yield (AY)
$BnEt_3NCl$	97 (n = 3)	60 ± 13 (n = 3)	65 ± 9 (n = 3)	71 ± 9 (n = 3)	52 ± 2 (n = 3)
Et_4NHCO_3	95 (n = 2)	41 ± 35 (n = 2)	45 ± 33 (n = 2)	54 ± 30 (n = 2)	33 ± 23 (n = 2)
$BnBu_3NCl$	97 ± 2 (n =3)	62 ± 2 (n = 3)	68 ± 1 (n = 3)	73 ± 2 (n = 3)	55 ± 1 (n = 3)

Table S2. Mean values (±SD) standard deviation of ^{18}F-elution, radiochemical conversions (RCC) and activity yields of [^{18}F]**12** using different salts. The corresponding concentrations of the different salt are as follows: Et_4NHCO_3 (1.0 mg, 5.2 µmol), $BnEt_3NCl$ (3.0 mg,13.1 µmol), and $BnBu_3NCl$ (3.0 mg, 9.6 µmol).

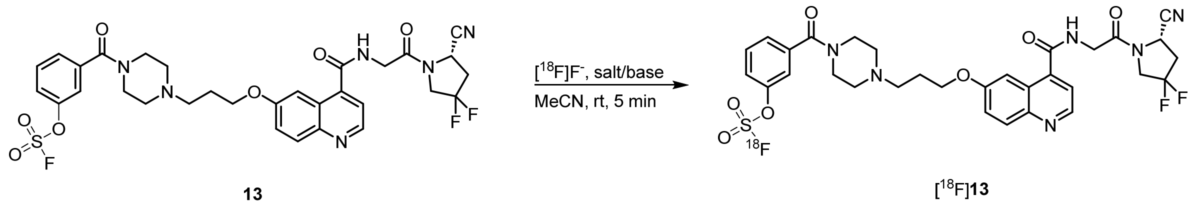

Salt	Run	^{18}F-Elution (%)	RCC (%) 30 sec	RCC (%) 1 min	RCC (%) 5 min	Activity Yield (AY)
$BnEt_3NCl$	**1**	94	46	47	63	35
	**2**	96	43	48	60	36
	**3**	97	42	50	61	36
Et_4NHCO_3	**4**	89	23	29	50	26
	**5**	93	30	37	46	29
$BnBu_3NCl$	**6**	97	48	50	57	44
	**7**	96	51	52	55	45
	**8**	96	32	31	38	39

Table S3. ^{18}F-Elution, radiochemical conversions (RCC) and activity yields of [^{18}F]**13** using different salts.

Salt	^{18}F-Elution (%)	RCC (%) 30 sec	RCC (%) 1 min	RCC (%) 5 min	Activity Yield (AY)
$BnEt_3NCl$	96 ± 2 (n = 3)	44 ± 2 (n = 3)	48 ± 2 (n = 3)	61 ± 2 (n = 3)	36 ± 1 (n = 3)
Et_4NHCO_3	91 ± 3 (n = 2)	27 ± 5 (n = 2)	33 ± 6 (n = 2)	48 ± 3 (n = 2)	28 ± 2 (n = 2)
$BnBu_3NCl$	96 ± 1 (n = 3)	44 ± 10 (n = 3)	44 ± 12 (n = 3)	50 ± 10 (n = 3)	43 ± 3 (n = 3)

Table S4. Mean values (±SD) standard deviation of ^{18}F-elution, radiochemical conversions (RCC) and activity yields of [^{18}F]**13** using different salts. The corresponding concentrations of the different salt are as follows: Et_4NHCO_3 (1.0 mg, 5.2 µmol), $BnEt_3NCl$ (3.0 mg,13.1 µmol), and $BnBu_3NCl$ (3.0 mg, 9.6 µmol).

Run	Precursor Quantity (mg)	RCC (%) 30 s	RCC (%) 1 min	RCC (%) 5 min
**1**	0.01	8	28	26
**2**	0.01	0	4	11
Mean value (±SD) Standard Deviation	0.01	4 ± 6 (n = 2)	16 ± 17 (n = 2)	19 ± 11 (n = 2)

Table S5. Reaction conditions as described in Materials and Methods section: $BnBu_3NCl$ was utilized as the PTA, and RCC of [^{18}F]**12** was analyzed using radio-UHPLC.

Radio-HPLC and Radio-UHPLC Chromatograms

HPLC & UHPLC experiments were performed according to the specified gradients outlined in the materials and methods section in the manuscript.

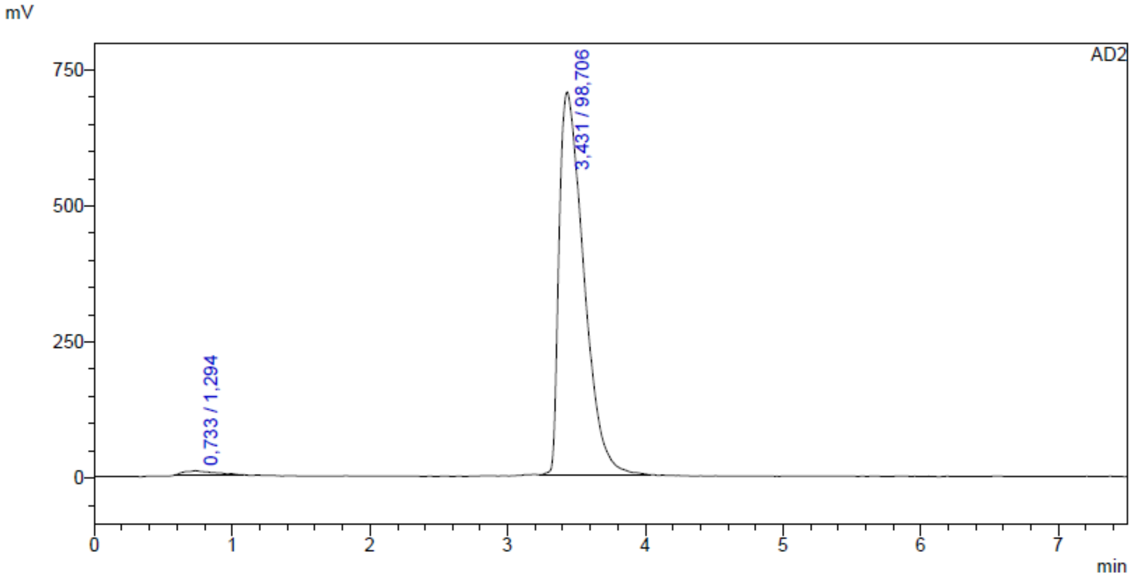

Figure S11. [^{18}F]**12** (radio-UHPLC) following SPE purification (Gradient A).

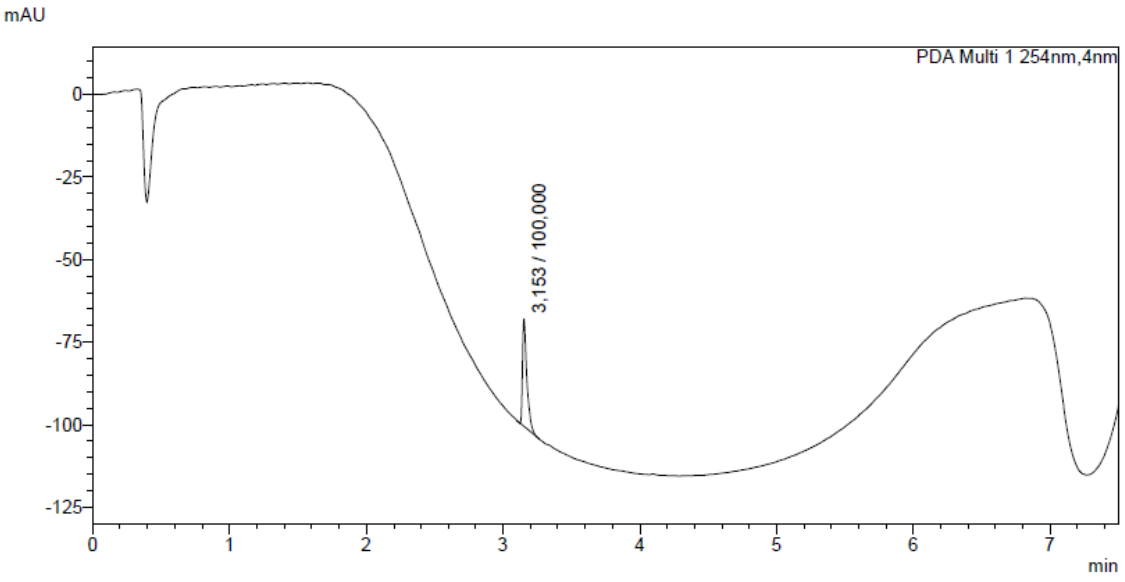

Figure S12. [^{19}F]**12** (UV, UHPLC) following SPE purification (Gradient A).

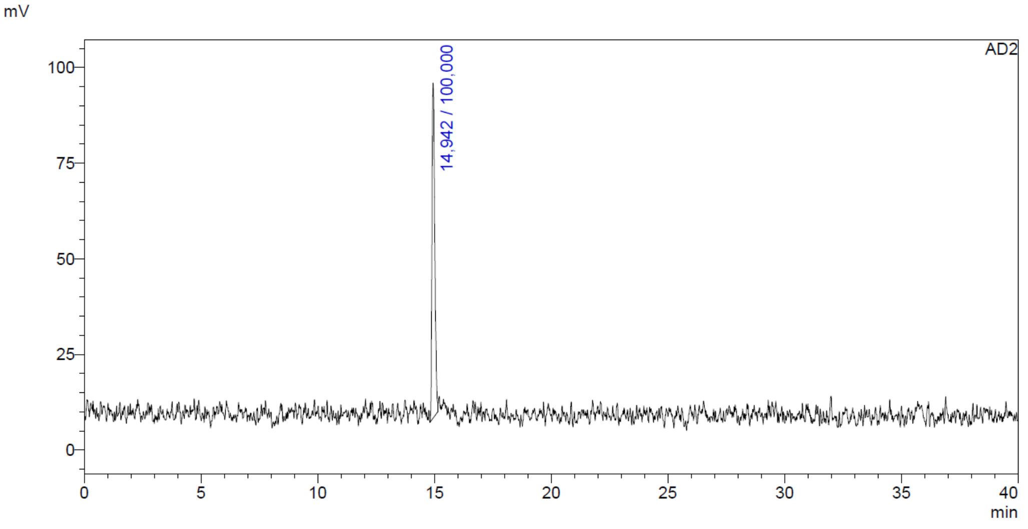

Figure S13. [^{18}F]**12** (radio-HPLC) following SPE purification (Gradient B).

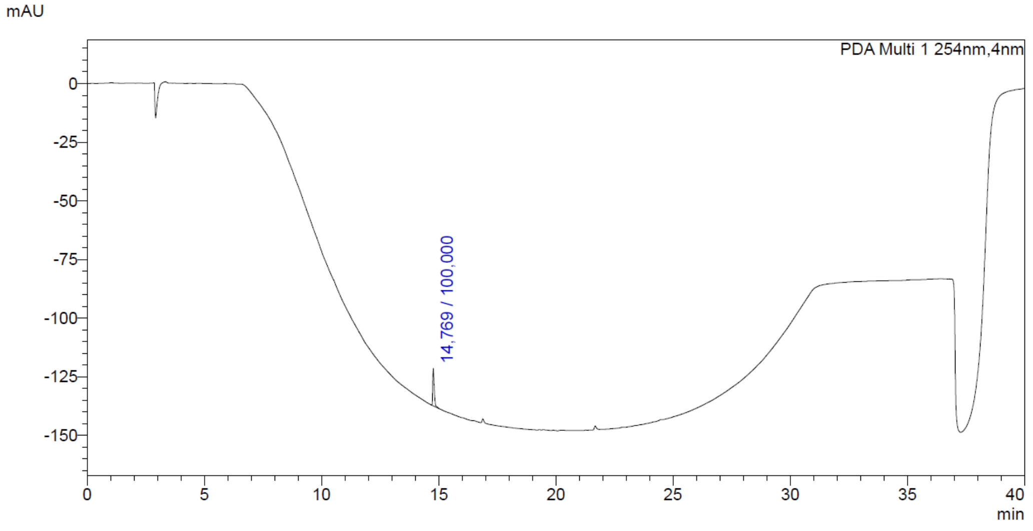

Figure S14. [^{19}F]**12** (UV, UHPLC) following SPE purification (Gradient B).

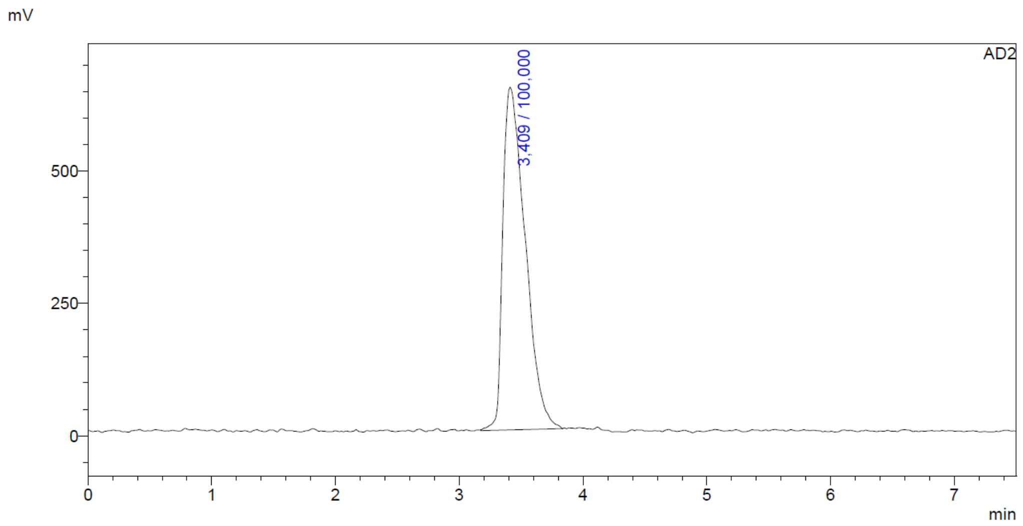

Figure S15. [^{18}F]**13** (radio-UHPLC) following SPE purification (Gradient A).

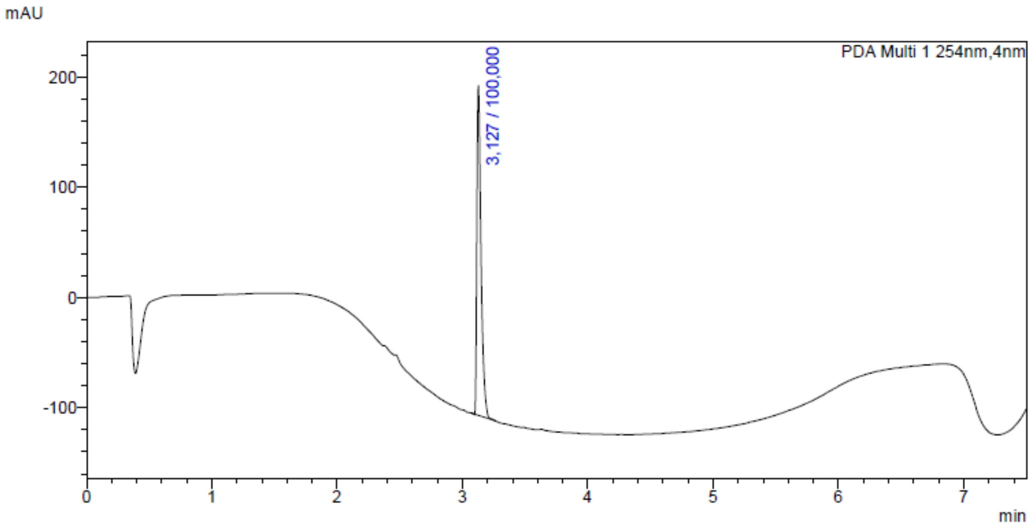

Figure S16. [^{19}F]**13** (UV, UHPLC) following SPE purification (Gradient A).

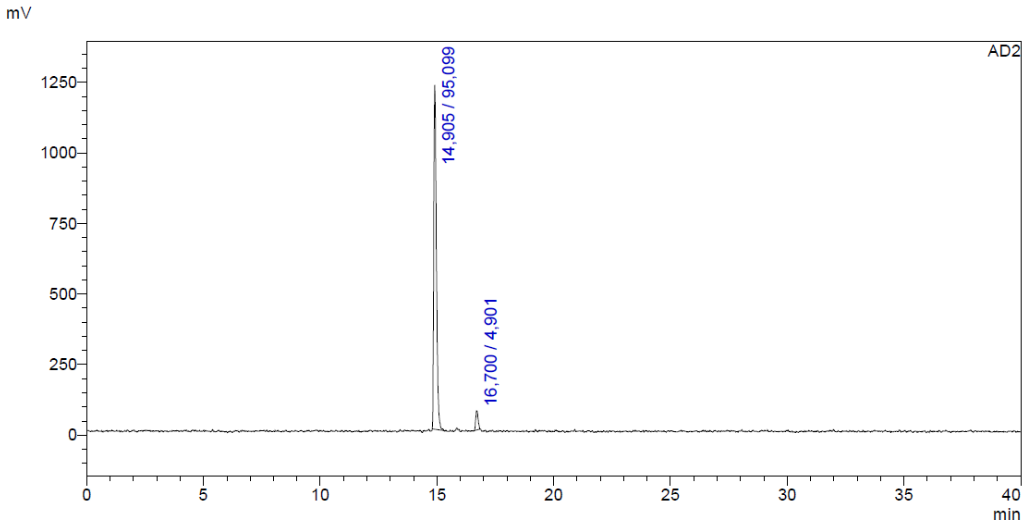

Figure S17. [^{18}F]**13** (radio-HPLC) following SPE purification (Gradient B).

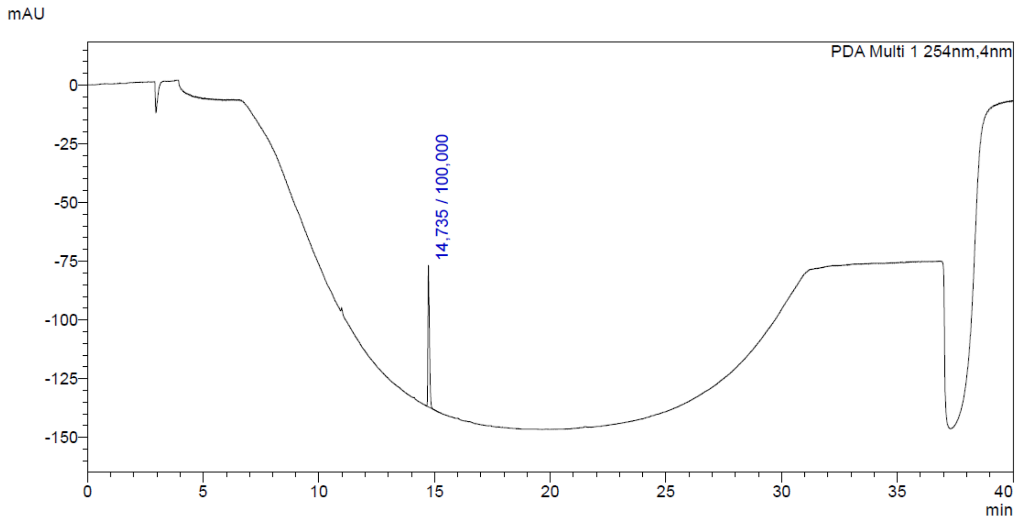

Figure S18. [18F]**13** (UV, HPLC) following SPE purification (Gradient B).

Stability Studies

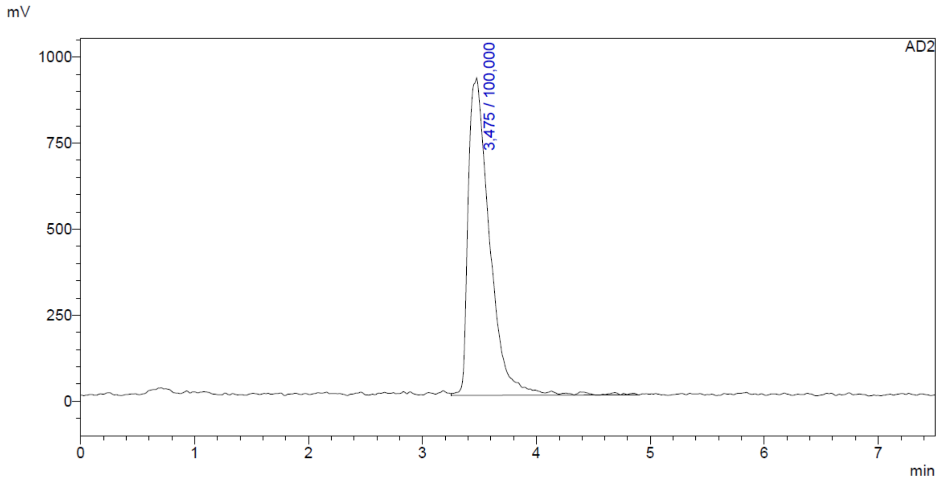

Figure S19. [18F]**12** (radio-UHPLC) following 120 min incubation in PBS buffer (pH 7,4) (Gradient A).

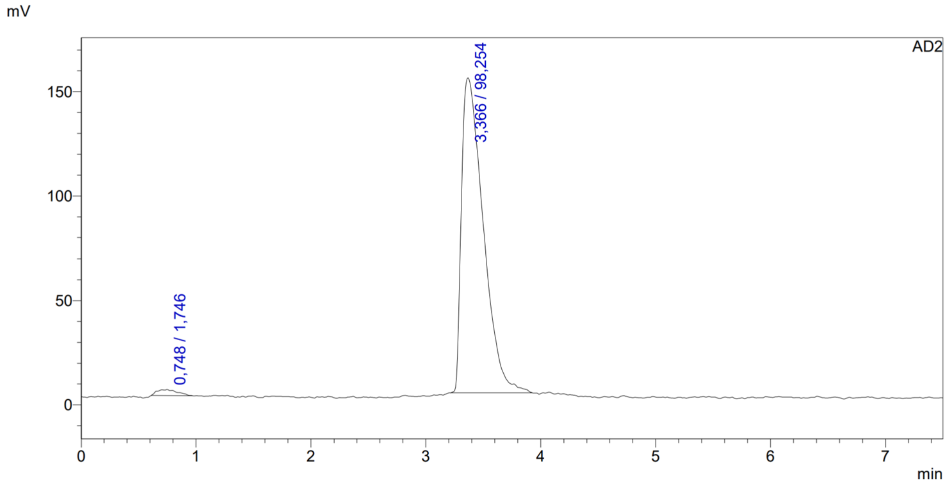

Figure S20. [18F]**13** (radio-UHPLC) following 120 min incubation in PBS buffer (pH 7,4) (Gradient A).

Determination of Molar Activity

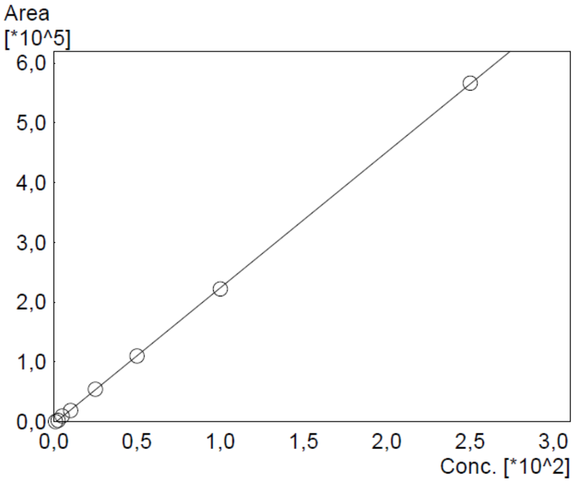

Figure S21. Calibration curve for molar activity determination of [^{18}F]**12**.

Calculation: A small aliquot of 6.80 MBq of [^{18}F]**12** in EtOH (10 µL) was added to 200 µL sample (1:1, MeCN/H_2O). Injection of 3 µL (315 KBq) of the [^{18}F]**12** sample for UHPLC analysis was performed in triplicate using an optimized gradient used to construct the calibration curve (Fig. S9). The mean peak area of the corresponding non-radioactive reference compound [^{19}F]**12** observed in the UV channel was plotted along the calibration curve and the concentration of [^{19}F]**12** was found to be 5.8913 ± 0.46 pmol (on column). Accordingly, the A_m value for [^{18}F]**12** was determined to be 53 ± 2 GBq/µmol, from a starting activity of 47.4 GBq with a radiolabeling precursor concentration of 0.145 µmol (0.1 mg of [^{19}F]**12**).

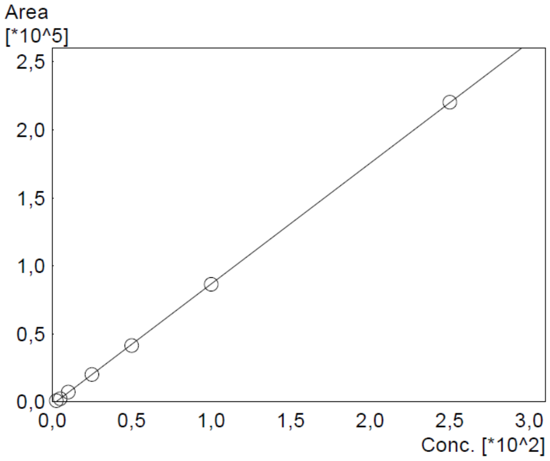

Figure S22. Calibration curve for molar activity determination of [^{18}F]**13**.

Calculation: A small aliquot of 13.35 MBq of [^{18}F]**13** in EtOH (10 µL) was added to 200 µL sample (1:1, MeCN/H_2O). Injection of 8 µL (509 KBq) of the [^{18}F]**13** sample for UHPLC analysis was performed in triplicate using an optimized gradient used to construct the calibration curve (Fig. S9). The mean peak area of the corresponding non-radioactive reference compound [^{19}F]**13** observed in the UV channel was plotted along the calibration curve and the concentration of [^{19}F]**13** was found to be 22,521 pmol (on column). Accordingly, the A_m value for [^{18}F]**13** was determined to be 22.58 GBq/µmol, from a starting activity of 9.3 GBq with a radiolabeling precursor concentration of 0.145 µmol (0.1 mg of [^{19}F]**13**).

Liver Microsome Experiments

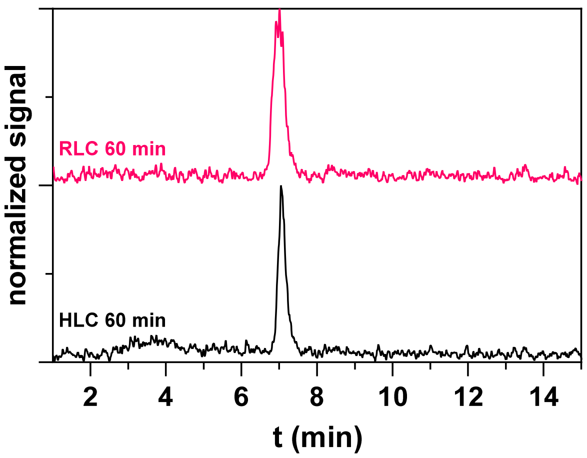

Figure S23. Radioactivity-detected HPLC chromatograms of [^{18}F]**12** after incubation with human and rat liver cytosol for 60 min. No degradation of [^{18}F]**12**, which has a retention time of 7.0 min, was observed. Conditions: 10 mM PBS (pH 7.4), 2 mg/mL HLC or RLC, 21 MBq/mL or 2.1 µM [^{18}F]**12**, 1% EtOH (v/v).

Composition of Supersol

**Supersol** (100 mL)					
Detergents	Quantity		Final Concentration		
EtOH	20	mL	20	%	
Triton X-100	0.5	mL	0.5	%	
EDTA	5	mL	5	mM	5 mL with 100 mM solution.
o-Phenanthroline	50	µL	0.5	mM	50 µL with 1 M solution.
Saponine	100	mg	0.1	%	

Table S6. Composition of Supersol solubilizing agent.

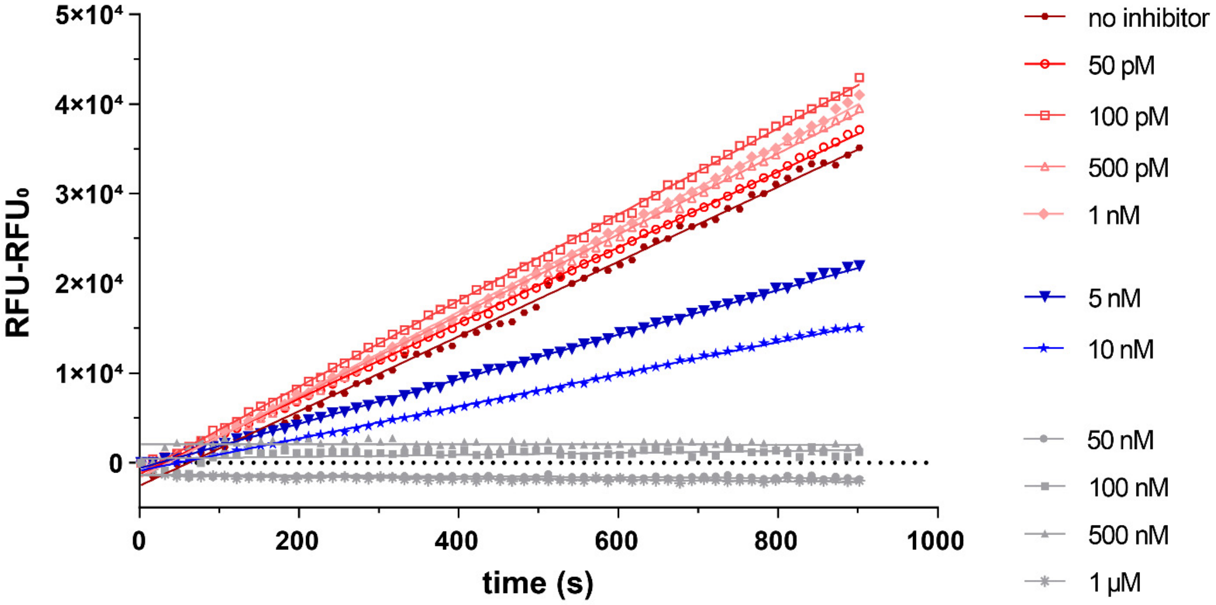

Figure S24. Linearity of fluorescence increase, catalyzed by human recombinant FAPα processing of a peptide substrate (Ala-Pro-AMC dipeptide).

References

(1) Jansen, K.; Heirbaut, L.; Verkerk, R.; Cheng, J. D.; Joossens, J.; Cos, P.; Maes, L.; Lambeir, A.-M.; De Meester, I.; Augustyns, K.; Van Der Veken, P. Extended Structure–Activity Relationship and Pharmacokinetic Investigation of (4-Quinolinoyl)Glycyl-2-Cyanopyrrolidine Inhibitors of Fibroblast Activation Protein (FAP). *J. Med. Chem.* **2014**, *57* (7), 3053–3074. https://doi.org/10.1021/jm500031w.

(2) Lindner, T.; Loktev, A.; Altmann, A.; Giesel, F.; Kratochwil, C.; Debus, J.; Jäger, D.; Mier, W.; Haberkorn, U. Development of Quinoline-Based Theranostic Ligands for the Targeting of Fibroblast Activation Protein. *J Nucl Med* **2018**, *59* (9), 1415–1422. https://doi.org/10.2967/jnumed.118.210443.

(3) Beerkens, B. L. H.; Wang, X.; Avgeropoulou, M.; Adistia, L. N.; Van Veldhoven, J. P. D.; Jespers, W.; Liu, R.; Heitman, L. H.; IJzerman, A. P.; Van Der Es, D. Development of Subtype-Selective Covalent Ligands for the Adenosine A_{2B} Receptor by Tuning the Reactive Group. *RSC Med. Chem.* **2022**, *13* (7), 850–856. https://doi.org/10.1039/D2MD00132B.
